# Whether we win or with whom we watch: How international sporting events link to life satisfaction and work motivation

**DOI:** 10.1111/bjso.70115

**Published:** 2026-08-07

**Authors:** Meikel Neumann, Annik Strauch, Lea Boecker, Kathi Diel, Yannik A. Escher, Paweł Muniak, Danna Oomen, Hannes M. Petrowsky, Leonie Schwarz, Marcel Weber, Malte Friese, Oliver Genschow, S. Alexander Haslam, David D. Loschelder

**Affiliations:** ^1^ School of Management and Technology Leuphana University Lüneburg Lüneburg Germany; ^2^ Department of Psychology Saarland University Saarbrücken Germany; ^3^ Department of Social Psychology SWPS University of Social Sciences and Humanities Warsaw Poland; ^4^ School of Psychology University of Queensland Brisbane Queensland Australia

**Keywords:** experience sampling, international sporting events, life satisfaction, social context, social identity, work motivation

## Abstract

International sporting events like the UEFA EURO 2024 provide a powerful natural context to study how social identity dynamics and social processes link to well‐being and motivation beyond the sporting domain. In this pre‐registered longitudinal experience‐sampling study (*N* = 842; *k* = 2,414 post‐game responses) across five European countries, we examined how social context (watching alone vs. with others) and game result (win vs. loss) link to spectators' life satisfaction and work motivation. Drawing on social identity and self‐categorisation theory, we explored mediating processes including team, football, and national identity, as well as social processes—social connectedness and game‐related socialising. Multilevel analyses revealed that watching games with others was unexpectedly associated with lower work motivation, an effect explained by alcohol consumption. The social context was unrelated to life satisfaction. Game result (i.e., a win) was positively related to life satisfaction and work motivation, mediated by social identities and social processes. These findings demonstrate that collective success—rather than shared spectating—coincides with positive psychological spill‐overs from leisure to work. By situating these effects within a social identity framework, the study highlights how collective experiences relate to both evaluative well‐being (i.e., life satisfaction) and motivational outcomes (i.e., work motivation) in everyday and organisational life.

Large‐scale international sporting events, such as the UEFA EURO 2024, the European Championship in football, attract massive global audiences and have consequences that extend far beyond the sport itself. Millions of individuals experience strong emotional reactions to these events, as they follow games for their suspense and dynamics (Unanue et al., 2022; Wann, 2006). The experience of such events is also inherently social, as they are often shared and discussed with others.

From a social identity perspective, such events create collective experiences that connect individuals to larger ingroups, be they fans, teams or nations. These events thus provide a fertile ground to examine how shared identities spill over to spectators' daily lives. Social identity theory (SIT; Tajfel & Turner, 1979) and self‐categorisation theory (Turner et al., 1994) suggest that these shared experiences activate collective self‐definitions, turning individual spectators into members of psychologically meaningful groups whose shared fate (winning or losing) shapes psychological outcomes (Haslam, 2004; Tajfel & Turner, 1979).

While social identity theory and self‐categorisation theory are closely related and jointly form the broader social identity approach, they have complementary features. Social identity theory outlines a process through which social categorisation and the situational salience of group membership precede social comparison and its psychological consequences. Complementing this, self‐categorisation theory specifies the context‐sensitive mechanisms through which social identities become salient. Together, both approaches describe a process in which contextual cues shape identities, which in turn have a range of downstream psychological and behavioural outcomes (Tajfel & Turner, 1979; Trepte & Loy, 2017).

Previous research suggests that both the sport itself (Jang et al., 2018; Unanue et al., 2022) as well as the social context (Neville & Reicher, 2011; Stieler & Germelmann, 2016) play important roles in determining how international sporting events are associated with individual, societal and economic outcomes. However, research that examines both sport‐related and social‐context‐related factors in combination remains scarce. Investigating their interplay promises to deliver new insights into how both *jointly* affect spectators' experiences.

With this goal in mind, we collected longitudinal data across five European countries during the four‐week UEFA EURO 2024 and investigated how both the social context of watching a game (i.e., watching alone vs. with others) and the game result (i.e., win vs. loss) were associated with individuals' life satisfaction and work motivation. Seeking to illuminate the underlying mechanisms, we examined multiple mediators, including identity‐based processes (i.e., team, football, and national identities) and social processes (i.e., social connectedness and game‐related socialising) that stem from shared group membership.

International sporting events typically attract widespread public attention (e.g., McGillivray, 2014; Mutz & Gerke, 2018). As such, these events may influence not only individuals' private lives but also more distal domains, including work life. Interestingly, previous research has primarily focused on either economic and societal outcomes (e.g., Lee Ludvigsen et al., 2022) or on individuals' subjective well‐being (e.g., Elling et al., 2014). Surprisingly few studies have explored how international sporting events influence individuals' working lives (cf. Gkorezis et al., 2016). The present study addresses this gap by exploring how major sporting events spill over into both private and work domains through the lens of social identity processes.

Relying on prior research, one can plausibly reason that international sporting events are connected to individuals' private and work lives in ways that are mediated by social identity processes (e.g., Jang et al., 2018; Kesler & Wann, 2020; Stieler & Germelmann, 2016; Wann & Branscombe, 1992), because such events provide contexts in which collective identities are activated, maintained, and made meaningful (Tajfel & Turner, 1979). Indeed, given the association between sport and social group dynamics, it is likely that the way individuals define themselves as part of a group (‘we’ rather than ‘I’) is central to understanding these effects (Tajfel & Turner, 1979; Turner et al., 1994).

Building on this framework, our research positions sporting events as powerful group‐level experiences that can strengthen or weaken collective identities and, in turn, influence well‐being and work motivation through what Haslam et al. (2018) describe as the ‘social cure’. In this perspective, group identification acts as a psychological resource, promoting belonging, connectedness, and purpose, which enhance both hedonic and eudaimonic outcomes. Conversely, when group settings encourage unhealthy behaviours (e.g., excessive drinking), these same dynamics can yield a ‘social curse’ (Haslam et al., 2018).

Against this background, the present research seeks to make a fourfold contribution to the literature. First, we extend prior research on international sporting events by integrating two fundamental but typically separate aspects of spectatorship into a single framework. Whereas prior work has largely examined either the game result, such as a win versus a loss (e.g., Jang et al., 2017, 2018; Stieger et al., 2015; Unanue et al., 2022), or the social context, such as shared spectatorship and collective participation (e.g., Inoue et al., 2017; Neville & Reicher, 2011; Stieler & Germelmann, 2016), our study considers both simultaneously. Second, we examine the prediction that identity‐based processes are relevant not only for evaluative well‐being, but also for work‐related motivation, thereby extending research on spill‐overs of sporting events beyond private life and into everyday functioning across life domains. Third, we develop a process account showing how collective experiences relate to downstream psychological outcomes via social identities and social processes such as feelings of social connectedness and game‐related socialising. Fourth, we provide a naturalistic and longitudinal test of these processes using experience‐sampling data collected across five countries during an international sports tournament, thereby offering ecologically valid evidence for within‐person fluctuations in identity salience and associated psychological outcomes.

## THEORETICAL BACKGROUND

### The impact of international sporting events

#### Collective effects – Economic and societal

International sporting events have significant economic and societal implications (e.g., Lee Ludvigsen et al., 2022). Economically, they generate revenue streams, ranging from tourism to sponsorships and advertising. Simultaneously, they require substantial investments in infrastructure and facilities, resulting in overall mixed economic outcomes (Baade & Matheson, 2004; Crompton, 2014; Horne, 2007; Kavetsos & Szymanski, 2010; Lee Ludvigsen et al., 2022). Similarly, societal effects are ambivalent. Sporting events can enhance social cohesion, inclusivity and diversity (e.g., Mair et al., 2023). Hosting a major event can temporarily boost public mood through a short‐lived ‘feel‐good factor’ (Kavetsos & Szymanski, 2010). At the same time, there is a dark side, as international sporting events can fuel nationalism, exclusion and exploitation (e.g., Mair et al., 2023).

#### Individual effects – Life satisfaction and work motivation

Alongside these collective effects, we posit that there are individual effects, particularly concerning life satisfaction and work motivation. Regarding life satisfaction, previous research has examined how major sporting events affect individuals' life satisfaction, using either the sport itself (i.e., the game result) or the social context (e.g., the shared viewing experience) as predictors. For instance, a national team win can temporarily enhance well‐being measures (Elling et al., 2014; Jang et al., 2017, 2018; Unanue et al., 2022). Other research has focused on social aspects such as live spectatorship (Inoue et al., 2017) and hosting status (Kavetsos & Szymanski, 2010; Pawlowski et al., 2014), all of which may positively relate to life satisfaction (Neville & Reicher, 2011). Here, we focus on life satisfaction as a cognitive evaluation of overall personal well‐being at a specific point in time (Baumeister et al., 2013; Diener et al., 1985).

From a social identity perspective, positive sporting outcomes may enhance life satisfaction because individuals internalise collective success as personally meaningful. Identification with teams and nations can foster belonging, collective pride and shared purpose, all of which are linked to well‐being (Haslam et al., 2018, 2020). Team identity, in particular, has been linked to enhanced well‐being because it provides a sense of belonging, shared purpose, and collective pride (Haslam et al., 2018, 2020). Belonging to valued groups satisfies psychological needs for meaning and connection that are central to both hedonic and eudaimonic well‐being. Conversely, a loss can lower well‐being because it threatens a valued social identity (Haslam et al., 2018, 2020).

Besides life satisfaction, the link between international sporting events and the work domain remains largely underexplored. Nevertheless, our primary hypothesis is that spectatorship may spill over into work life and, more specifically, that positive national team performance (i.e., wins) may be associated with higher levels of next‐day work motivation (see Gkorezis et al., 2016). In the present research, we conceptualise work motivation as a state‐level motivational readiness to engage in work‐related activities on a given day. This captures individuals' momentary willingness to invest effort and their general work energy (see Model of Human Energy; Quinn et al., 2012) rather than distinguishing between different motivational qualities (e.g., Deci & Ryan, 2000). Accordingly, our measure reflects a general activation of motivation in the work domain, capturing whether individuals feel more or less inclined to engage with their work on a given day. This unidimensional operationalisation in the present experience‐sampling context thus focuses on short‐term fluctuations in motivational activation.

Social identity theory provides a clear explanatory bridge for this spill‐over. When individuals' group identity is high and positive, for instance, after a win, collective pride can spill over to other domains. This strengthens motivation, energy and purpose at work, aligning with the broader concept of identity‐based motivation, where collective success enhances personal drive (Vo et al., 2022).

### Multiple underlying mechanisms – Social identities, socialising, and connectedness

The present research suggests that social identity processes are key to understanding how international sporting events affect individuals (Haslam, 2004; Haslam et al., 2018; Jang et al., 2018; Stieler & Germelmann, 2016). According to both social identity theory and self‐categorisation theory, the social context (e.g., watching a game alone vs. with others) should be associated with stronger group identities, such as team, football, and national identities. Watching a game with others should heighten identification, reinforcing group identity and self‐categorisation in terms of shared ingroup membership. In turn, this should be linked to a greater social connectedness and game‐related social behaviour.

We conceptualise this process as an identity‐based pathway, in which belonging to valued groups promotes well‐being and (work) motivation (Haslam et al., 2018). Specifically, the ‘social cure’ perspective (Haslam et al., 2018; Jetten et al., 2012) posits that group identity contributes to health and well‐being by enhancing perceived social support, belonging, and control. We thus conceptualise social connectedness and game‐related socialising as key components of the social cure pathway.

Importantly, these processes can be understood as sequentially linked: Contextual factors (e.g., watching with others or experiencing a collective win) may strengthen social identification, which in turn relates to greater social connectedness, increased socialising, and ultimately higher life satisfaction and work motivation.

In contrast, social identity theory also predicts that these same identity processes can produce costs when group norms encourage detrimental behaviour. In such cases, social engagement can turn into a ‘social curse’ (Haslam et al., 2018). For instance, social drinking after games may strengthen bonds but undermine next‐day work motivation.

**FIGURE 1:**
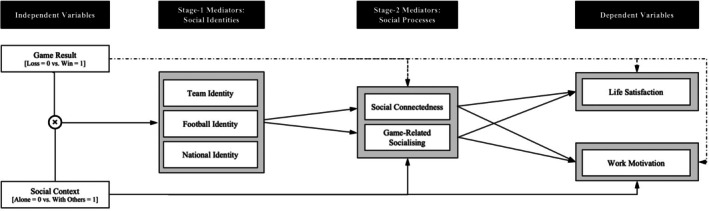
Proposed conceptual model. This conceptual model explores the effects of winning (vs. losing) and watching with others (vs. alone) on spectators' life satisfaction and work motivation, mediated by different identity‐based psychological mechanisms. Solid lines display the relationship between social context and life satisfaction as well as work motivation as a function of social identities and social processes, while the dashed lines (top) display the explored effects of game results.

Against this background and as indicated in Figure 1, we hypothesise that the social context of watching a game with others (vs. alone) is associated with increased shared social identities (i.e., team, football, and national identity), which in turn coincides with greater social connectedness and game‐related socialising, and ultimately with higher life satisfaction and work motivation (Haslam et al., 2018; Jetten et al., 2012; Tajfel & Turner, 1979; Turner et al., 1994).

We further hypothesise that the outcomes of international sporting events, specifically a win (vs. loss), are positively associated with life satisfaction and work motivation, both directly and indirectly via increased identity salience and subsequent social processes (e.g., Jang et al., 2018; Stieger et al., 2015; Unanue et al., 2022). Our model includes not only indirect effects through social identities and social processes but also direct effects of game result on life satisfaction and work motivation. We explore a possible interaction between social context and game result.

## OVERVIEW OF THE PRESENT RESEARCH

Our study used data that were collected as part of a larger research project that was pre‐registered on the Open Science Framework (see Petrowsky* et al., 2026). Data pertaining to the present research question have not been reported elsewhere.

### Transparency and openness

We pre‐registered the study's design, hypotheses, and analysis plan (see OSF). In line with the gatekeeping process described in the full project, data remained with the designated data officer until after the respective pre‐registrations were uploaded. All data, analysis code and supplementary materials are publicly available on OSF (see OSF). Data were analysed using *R*, version 4.4.0 (R Core Team,. 2024).

### Design and data collection

Data collection involved multiple measurement waves in an event‐sampling approach across five different countries during the UEFA EURO 2024, hosted in Germany from June 14th to July 14th, 2024. Our multi‐level design entails single data points across multiple measurement waves (Level 1), which are nested in individual participants (Level 2), and can be examined as a function of the respective national team winning, losing, or drawing the game the day before as well as the social context. We describe all items and variables that are relevant to the present study below (for details about the larger project, measurement waves, data collection procedure and verbatim measures, see Petrowsky* et al., 2026).

### Sample

We recruited a total of *N* = 1,012 participants from (*alphabetically*): England (*n* = 202), Germany (*n* = 204), Italy (*n* = 201), the Netherlands (*n* = 204) and Poland (*n* = 201). Participants who did not take part in at least one event‐specific questionnaire were excluded from the analyses (*n* = 60). On average, participants completed four event questionnaires (median response rate). As we focus on wins vs. losses, we excluded draws (*n* = 22) and participants who did not watch the game fully (*n* = 88). This way, we derived a final sample of 842 individuals (*M*_age_ = 33.53, *SD*_age_ = 10.47, range: 18–74 years): 574 (68.17%) identified as male, 266 (31.59%) as female, and 2 (0.24%) identified as diverse.

### Procedure and remuneration

Participants were recruited via Prolific, were 18 years or older, were residents of one of the five participating countries, and planned to follow the UEFA EURO 2024 tournament. Before the tournament, they completed an intake questionnaire that collected demographic information and assessed the outcome variables (i.e., to establish a pre‐tournament baseline). During the tournament, participants completed event‐specific questionnaires the day after every match that their national team played (i.e., after a win, loss, or draw).

Participants received monetary compensation for the intake questionnaire and for every completed event‐specific questionnaire with the chance of additional bonus payments in case of regular participation (see OSF for details).

### Measurement

#### Predictors

Our study focused on two key predictors, social context and game result. Social context served as our first predictor, coded as having watched the game with others (1) or alone (0). Of the initially recruited *k* = 3,627 event questionnaires reported the day after the game, about one quarter stated having watched the game at home alone (*k*_home alone_ = 899; 24.8%), while the rest stated having watched the game with others (*k*_with others_ = 2,176; 60.0%; e.g. at home or in the stadium). We excluded questionnaires in which participants reported having only watched parts of the game (*k*_watched parts of game_ = 481; 13.3%) and having not watched the game (*k*_did not watch_ = 71; 2.0%).

Game result served as our second predictor, coded as a win (1) or a loss (0). Of the *k* = 3,627 event questionnaires, about half were completed after a win (*k*_win_ = 1,704; 47.0%), while roughly one third were completed after a loss (*k*_loss_ = 1,132; 31.2%). We excluded those questionnaires, a fifth, that were completed after a draw (*k*_draw_ = 791; 21.8%).

In sum, the final amount of event questionnaires included for analyses was *k* = 2,414 (from *N* = 842).

#### Outcome variables

We assessed (1) life satisfaction (‘I am satisfied with my life today’; 7‐point Likert scale, 1 = *very dissatisfied* to 7 = *very satisfied*), and (2) work motivation (‘How motivated are you to work today?’; 1 = *not at all motivated* to 7 = *extremely motivated*) as outcome variables. To reduce participant burden in this experience‐sampling design, we deliberately used single‐item measures for all focal constructs (Gabriel et al., 2019). Prior research suggests that single‐item measures are appropriate for clearly defined, unidimensional constructs in repeated assessments (Cheung & Lucas, 2014; Matthews et al., 2022). Moreover, evidence from the Single‐Item Social Identification measure (SISI) indicates that such a measure can have good reliability and validity, particularly when capturing context‐specific fluctuations in identities (Postmes et al., 2013).

#### Underlying psychological mechanisms

To illuminate the underlying psychological mechanisms, we pre‐registered and assessed three Stage‐1 mediators: (1) *Team identity* (‘How strongly do you see yourself as a fan of the [country] national team today?’), (2) *football identity* (‘How strongly do you see yourself as a football fan today?’; both on a 7‐point scale: 1 = *not a fan at all*, 7 = *die‐hard fan*), and (3) *national identity* (‘How important is being [nationality] for you today?’; 1 = *not important at all*, 7 = *extremely important*).

Our conceptual model (Figure 1) proposed that these social identification processes would be associated with subsequent Stage‐2 mechanisms. Accordingly, we assessed two Stage‐2 mediators: (1) *social connectedness* (‘I feel socially connected with the world around me today’; 1 = *strongly disagree*, 7 = *strongly agree*) and (2) *game‐related socialising* (‘Since the end of yesterday's game, I was keen to talk to other people about this game’; 1 = *not at all willing*, 7 = *extremely willing*).

### Statistical models and analytic approach

For all pre‐registered hypotheses, we ran generalised linear mixed models (GLMM) with individual observations (Level 1) nested in participants (Level 2) using the *lme4* package (Bates et al., 2015). In line with best practice in multi‐level modelling (Enders & Tofighi, 2007; Yaremych et al., 2023), we centred all predictors and mediators (Level‐1, e.g. social context, game result, team identity, social connectedness) at the person mean.

We specified two alternative models for all analyses (see OSF): (1) a random‐intercept (RI) model that allows intercepts to vary randomly across participants, and (2) a random‐intercept plus random‐slope (RS) model that allows both intercepts and slopes to vary randomly across participants.^1^ To evaluate the two models, we compared their fit using likelihood‐ratio tests (*χ* ^2^) and model‐fit scores (AIC, BIC). Additionally, we explored mediation effects using the mediation package (Tingley et al., 2014) and the *bruceR* package (Bao, 2024) with 1000 Markov Chain Monte Carlo (MCMC) samples for effect and confidence interval (CI) estimation as well as a path analysis to test the sequential mediation simultaneously.

## RESULTS

We first examined the link between watching games together (vs. alone) and spectators' work motivation and life satisfaction. Subsequently, we report mediation analyses that examine underlying psychological processes (see Figure 1). We display descriptives in Table 1 and intercorrelations on both between‐ and within‐participant levels in Table 2.^2^

**TABLE 1:** Frequency and descriptive statistics for all variables.

Variable	Overall	Game result		Social context				
		Loss	Win	Alone	With others			
	*N* (%)	*N* (%)	*N* (%)	*N* (%)	*N* (%)			
1. Game result								
Loss = 0	946 (39.2%)	—	—	261 (40.2%)	685 (38.8%)			
Win = 1	1468 (60.8%)	—	—	389 (59.8%)	1079 (61.2%)			
2. Social context								
Alone = 0	650 (26.9%)	261 (27.6%)	389 (26.5%)	—	—			
With others = 1	1764 (73.1%)	685 (72.4%)	1079 (73.5%)	—	—			
	***M* (*SD*)**	***M* (*SD*)**	***M* (*SD*)**	***M* (*SD*)**	***M* (*SD*)**	***SD***_**person**_	***SD***_**games**_	**ICC**
3. Team identification	4.92 (1.40)	4.56 (1.56)	5.15 (1.24)	4.87 (1.47)	4.94 (1.38)	1.10	0.92	.59
4. Football identification	4.96 (1.47)	4.75 (1.57)	5.10 (1.38)	4.91 (1.54)	4.98 (1.44)	1.27	0.83	.70
5. National identification	4.41 (1.60)	4.39 (1.62)	4.42 (1.59)	4.33 (1.67)	4.44 (1.57)	1.36	0.87	.71
6. Social connectedness	4.47 (1.38)	4.26 (1.43)	4.60 (1.33)	4.24 (1.46)	4.55 (1.35)	0.97	1.00	.48
7. Game‐related socialising	4.65 (1.68)	4.14 (1.79)	4.97 (1.51)	4.51 (1.81)	4.70 (1.63)	1.01	1.35	.36
8. Life satisfaction	4.82 (1.39)	4.58 (1.40)	4.97 (1.35)	4.55 (1.46)	4.92 (1.35)	1.10	0.84	.63
9. Work motivation	4.02 (1.77)	3.83 (1.73)	4.13 (1.80)	4.06 (1.75)	4.00 (1.78)	1.13	1.37	.41
10. Alcohol consumption	1.37 (1.97)	1.26 (1.89)	1.44 (2.02)	0.75 (1.44)	1.60 (2.09)	1.40	1.39	.50

*Note*: *k* = 2414 observations from 842 participants. *M* (*SD*) = mean and standard deviation; *n* (%) = frequency and percentage for binary variables. *SD*_person_ = between‐person SD; *SD*_games_ = within‐person SD (null multilevel models). Dashes (—) indicate not applicable values.

Abbreviation: ICC, intraclass correlation coefficient.

**TABLE 2:** Intercorrelations for all variables at both within‐ and between‐level.

Variable	1.	2.	3.	4.	5.	6.	7.	8.	9.	10.
1. Game result (win = 1)		.06	.12***	.01	−.08*	.02	.05	.08*	.02	.14***
2. Social context (with others = 1)	−.02		.05	.04	.08*	.16***	.13***	.19***	.06	.19***
3. Team identification	.34***	.02		.73***	.54***	.39***	.47***	.25***	.27***	.04
4. Football identification	.26***	.01	.53***		.41***	.39***	.51***	.25***	.22***	.01
5. National identification	.10***	−.03	.34***	.28***		.41***	.34***	.28***	.32***	.02
6. Social connectedness	.19***	.02	.29***	.24***	.20***		.45***	.58***	.44***	−.06
7. Game‐related socialising	.38***	−.01	.34***	.35***	.23***	.29***		.34***	.27***	.01
8. Life satisfaction	.24***	−.00	.26***	.26***	.20***	.37***	.29***		.41***	−.01
9. Work motivation	.13***	−.09***	.21***	.22***	.13***	.25***	.20***	.28***		−.13***
10. Alcohol consumption	.01	.20***	.01	−.01	.00	.02	−.01	.00	−.16***	

*Note*: Intercorrelations are displayed based on *k* = 2,414 observations from *N* = 842 participants. Below the diagonal are correlations at the game level (within‐level). Above the diagonal are correlations at the person level (between‐level). Statistically significant bivariate correlations are displayed with asterisks.

* *p* < .05;

*** *p* < .001.

### Social context – Watching alone vs. with others

#### Direct effects

We first examined the relationship between social context and work motivation.^3^ For life satisfaction, neither the RI model, *B* = −0.005, *SE* = 0.06, *t*(1582.00) = −0.08, *p* = .934, nor the RS model, *B* = −0.004, *SE* = 0.06, *t*(180.97) = −0.06, *p* = .952, were significant. The model comparison indicated no improvement in fit with random slopes included, *χ* ^2^(2) = 0.57, *p* = .751. Deviance was marginally lower in the more complex model (7,425.50 vs. 7,426.00), but information criteria favoured the simpler model (AIC_RI_ = 7,434.00 vs. AIC_RS_ = 7,437.50; BIC_RI_ = 7,457.20 vs. BIC_RS_ = 7,472.20).

For work motivation, social context was a significant predictor in the RI model, *B* = −0.38, *SE* = 0.10, *t*(1,617.30) = −3.64, *p* < .001. The RS model produced nearly identical estimates, *B* = −0.38, *SE* = 0.10, *t*(1,600.71) = −3.63, *p* < .001 (see Table 3a for an overview). As the direct effect was negative, this indicates that watching with others (vs. alone) coincided with *lower* work motivation the day after the game. Deviance was slightly lower for the RS model (9,210.70 vs. 9,211.10), AIC favoured the more complex RS model (AIC_RI_ = 9,211.10 vs. AIC_RS_ = 9,210.70), while BIC favoured the simpler RI model (BIC_RI_ = 9,242.20 vs. BIC_RS_ = 9,257.40). Model fit statistics indicated no significant improvement when including random slopes, *χ* ^2^(2) = 0.41, *p* = .815.

**TABLE 3A:** Summary of direct and indirect effects of social context via alcohol consumption on work motivation.

Outcome	Path	*B*	*SE*	CI_95%_	*p*
Work motivation	Social context → Work motivation	−0.38	0.10	[−0.59, −0.18]	< .001
	Controlling for alcohol consumption	−0.26	0.10	[−0.46; −0.06]	.012
	Via alcohol consumption	−0.12	0.02	[−0.17, −0.08]	< .001

*Note*: This table summarises key direct and indirect effects of social context (watching with others vs. alone) on work motivation. Indirect effects are based on mediation analyses. Estimates are based on the random‐intercept (RI) models.

#### Alcohol consumption as underlying mechanism for less work motivation

Alcohol consumption was included as part of the original data collection and assessed in the same event‐specific questionnaire as the other variables (i.e., on the day following the respective game). While we did not preregister alcohol consumption as a mediator, we used it to explore its link in light of the unexpected negative association between watching with others and reduced next‐day work motivation.

To that end, we used *alcohol consumption* as an underlying mechanism (i.e., ‘How much alcohol did you drink during or after yesterday's game?’; 1 = *very little*, 7 = *very much*, alternative: *‘I did not drink alcohol’*; see Howell et al., 2014). We found an indirect effect in that alcohol consumption mediated the link from watching with others on impaired work motivation the next day, *B* = −0.12, *SE* = 0.02, *p* < .001, CI_95%_ [−0.17, −0.08] (direct effect; *B* = −0.26, *SE* = 0.10, *p* = .012, CI_95%_ [−0.46, −0.06]). This suggests that individuals who watched the game with others consumed more alcohol on match day, which in turn coincided with them having lower work motivation the next day.^4^

### Game result—Win vs. loss

#### Direct effects

For life satisfaction, game result (vs. loss) emerged as a significant predictor in the RI model, *B* = 0.42, *SE* = 0.04, *t*(1,580.00) = 9.90, *p* < .001. The RS model yielded similar results, *B* = 0.43, *SE* = 0.05, *t*(460.66) = 8.79, *p* < .001. Their team winning the day before coincided with individuals' reporting higher life satisfaction the next day. Model comparison revealed a better fit for the RS model including random slopes, *χ* ^2^(2) = 29.00, *p* < .001. Deviance was lower for the more complex RS than for the RI model (7,301.90 vs. 7,330.90; model fit: AIC_RS_ = 7,338.90 vs. AIC_RI_ = 7,362.00; BIC_RS_ = 7,348.60 vs. BIC_RI_ = 7,362.00).

For work motivation, game result (vs. loss) emerged as a significant predictor in the RI model, *B* = 0.36, *SE* = 0.07, *t*(1,619.00) = 5.05, *p* < .001, with nearly identical results for the RS model, *B* = 0.36, *SE* = 0.07, *t*(480.68) = 5.02, *p* < .001. Their team winning the day before coincided with individuals' reporting higher work motivation the following day. Model comparison showed no improvement for the RS model over the simpler RI model, *χ* ^2^(2) = 0.19, *p* = .908. Deviance was identical for both models (i.e., exactly 9,199.00 for both); however, the information criteria slightly favoured the simpler model (AIC_RI_ = 9,207.00 vs. AIC_RS_ = 9,210.80; BIC_RI_ = 9,230.10 vs. BIC_RS_ = 9,245.00).

#### Serial mediation of the full conceptual model

Next, we examined our full conceptual model with its serial mediation propositions, that is, a model in which social identity processes (Stage‐1 mediators) coincide with feelings of social connectedness as well as game‐related socialising (Stage‐2 mediators). We also explored the role of three different social identities (i.e., those related to the team, to football, and to the nation) in three separate serial mediation analyses (see Process Model 6; Hayes, 2018).

##### Team identity

**FIGURE 2:**
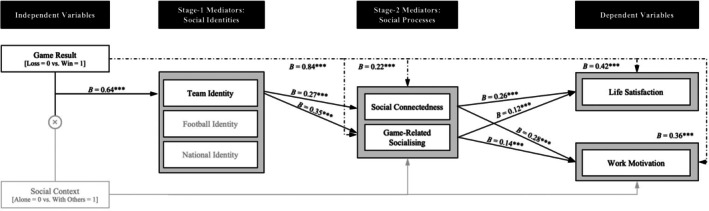
Serial mediation via team identity as Stage‐1 mediator. This shows a significant serial mediation with team identity as a Stage‐1 mediator. ***p < .001; **p < .01; *p < .05. For readability, only *B* values are displayed.

For life satisfaction (see Figure 2), the first set of serial mediations revealed a significant indirect effect of game result through team identity and subsequent social connectedness, *B* = 0.05, CI_95%_ [0.03, 0.06] (direct effect: *B* = 0.25, CI_95%_ [0.10, 0.40]). We also found an indirect effect via team identity and game‐related socialising, *B* = 0.02, CI_95%_ [0.01, 0.04] (direct effect: *B* = 0.22, CI_95%_ [0.07, 0.37]).

For work motivation, we found an indirect effect for game result via team identity and social connectedness, *B* = 0.05, CI_95%_ [0.03, 0.07] (direct effect: *B* = 0.12, CI_95%_ [−0.07, 0.31]). Similarly, we found an indirect effect via team identity and game‐related socialising, *B* = 0.03, CI_95%_ [0.02, 0.05] (direct effect: *B* = 0.06, CI_95%_ [−0.16, 0.27]; see Figure 2 and Table 3b).

##### Football identity

**FIGURE 3:**
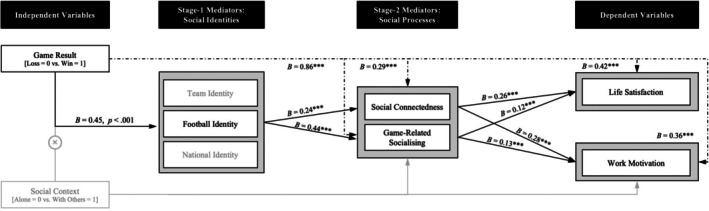
Serial mediation with football identity as Stage‐1 mediator. This shows a significant serial mediation with football identity as a Stage‐1 mediator. ***p < .001; **p < .01; *p < .05. For readability, only *B* values are displayed.

For life satisfaction (see Figure 3), serial mediation analyses revealed a significant indirect effect of game result via football identity and social connectedness, *B* = 0.03, CI_95%_ [0.02, 0.04] (direct effect: *B* = 0.25, CI_95%_ [0.11, 0.39]). We also found an indirect effect via football identity and game‐related socialising, *B* = 0.02, CI_95%_ [0.01, 0.04] (direct effect: *B* = 0.22, CI_95%_ [0.07, 0.37]).

For work motivation, we found an indirect effect via football identity and social connectedness, *B* = 0.03, CI_95%_ [0.02, 0.04] (direct effect: *B* = 0.25, CI_95%_ [0.11, 0.39]), and via football identity and game‐related socialising, *B* = 0.03, CI_95%_ [0.01, 0.04] (direct effect: *B* = 0.09, CI_95%_ [−0.11, 0.30]; see Figure 3 and Table 3b).

**TABLE 3B:** Summary of total and indirect effects of game result.

Outcome	Effect type	Path	*B*	CI_95%_
Life satisfaction	Total	Game result → Life satisfaction	0.42	[0.28, 0.56]
	Indirect	Via team identity → social connectedness	0.05	[0.03, 0.06]
	Indirect	Via team identity → game‐related socialising	0.02	[0.01, 0.04]
	Indirect	Via football identity → social connectedness	0.03	[0.02, 0.04]
	Indirect	Via football identity → game‐related socialising	0.02	[0.01, 0.04]
	Indirect	Via national identity → social connectedness	0.01	[0.005, 0.02]
	Indirect	Via national identity → game‐related socialising	0.007	[0.003, 0.01]
Work motivation	Total	Game result → Work motivation	0.36	[0.18, 0.53]
	Indirect	Via team identity → social connectedness	0.05	[0.03, 0.07]
	Indirect	Via team identity → game‐related socialising	0.03	[0.02, 0.05]
	Indirect	Via football identity → social connectedness	0.03	[0.02, 0.04]
	Indirect	Via football identity → game‐related socialising	0.03	[0.01, 0.04]
	Indirect	Via national identity → social connectedness	0.01	[0.005, 0.02]
	Indirect	Via national identity → game‐related socialising	0.01	[0.004, 0.02]

*Note*: Total effects reflect models without mediators. Indirect effects are based on serial mediation analyses.

##### National identity

**FIGURE 4:**
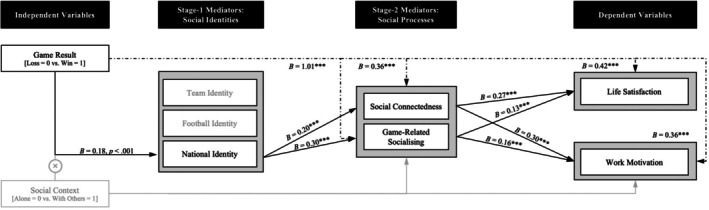
Serial mediation with national identity as a Stage‐1 mediator. This shows a significant serial mediation with national identity as a Stage‐1 mediator. ***p < .001; **p < .01; *p < .05. For readability, only *B* values are displayed.

For life satisfaction (see Figure 4), serial mediation revealed a significant indirect effect via national identity and subsequent social connectedness, *B* = 0.01, CI_95%_ [0.005, 0.02] (direct effect: *B* = 0.29, CI_95%_ [0.15, 0.44]). We also found serial mediation via national identity and game‐related socialising, *B* = 0.007, CI_95%_ [0.003, 0.01] (direct effect: *B* = 0.26, CI_95%_ [0.11, 0.41]).

For work motivation, analyses revealed a serial mediation via national identity and social connectedness, *B* = 0.01, CI_95%_ [0.005, 0.02] (direct effect: *B* = 0.21, CI_95%_ [0.03, 0.40]), and via national identity and game‐related socialising, *B* = 0.01, CI_95%_ [0.004, 0.02] (direct effect: *B* = 0.16, CI_95%_ [−0.05, 0.37]).

### Testing the three Stage‐1 mediators simultaneously

Simultaneous mediation analysis to complement the serial mediation analyses using the multilevel *sem* function of the *lavaan* package in R Studio (using uncentered variables, e.g. Lüdtke et al., 2008) replicated the identified pattern. We found significant indirect effects for team identity, football identity, and for national identity. The latter had much smaller effects, suggesting that the former identities are more important for explaining the relationship between game result and life satisfaction and work motivation compared to national identity.

#### Team and football identity

For both life satisfaction and work motivation, the model (see Table 4a) suggested significant sequential indirect effects via team identity and football identity via both social connectedness and game‐related socialising (ind_result_teamid_socialconn_lifesat_: *B* = 0.05, CI_95%_ [0.03, 0.06]; ind_result_teamid_gameresol_lifesat_: *B* = 0.03, CI_95%_ [0.01, 0.04]; ind_result_teamid_socialconn_workmotiv_: *B* = 0.05, CI_95%_ [0.03, 0.07]; ind_result_teamid_gameresol_workmotiv_: *B* = 0.03, CI_95%_ [0.02, 0.05]; ind_result_footballid_socialconn_lifesat_: *B* = 0.03, CI_95%_ [0.02, 0.04]; ind_result_footballid_gameresol_lifesat_: *B* = 0.02, CI_95%_ [0.01, 0.04]; ind_result_footballid_socialconn_workmotiv_: *B* = 0.03, CI_95%_ [0.02, 0.05]; ind_result_footballid_gameresol_workmotiv_: *B* = 0.03, CI_95%_ [0.01, 0.04]).

**TABLE 4A:** Summary of sequential indirect effects of game result (Win vs. Loss): Team and football identity.

Outcome	Stage‐1 mediator	Stage‐2 mediator	*B*	CI_95%_	Interpretation
Life satisfaction	Team identity	Social connectedness	0.05	[0.03, 0.06]	Significant
		Game‐related socialising	0.03	[0.01, 0.04]	Significant
Work motivation		Social connectedness	0.05	[0.03, 0.07]	Significant
		Game‐related socialising	0.03	[0.02, 0.05]	Significant
Life satisfaction	Football identity	Social connectedness	0.03	[0.02, 0.04]	Significant
		Game‐related socialising	0.02	[0.01, 0.04]	Significant
Work motivation		Social connectedness	0.03	[0.02, 0.05]	Significant
		Game‐related socialising	0.03	[0.01, 0.04]	Significant

*Note*: Indirect effects are based on serial mediation analyses (PROCESS Model 6; Hayes, 2018), with Stage‐1 mediators (team, football, or national identity) and Stage‐2 mediators (social connectedness and game‐related socialising). Unstandardised coefficients (*B*) are reported. Confidence intervals (CI_95%_) are based on bootstrapping; effects are statistically significant when the confidence interval does not include zero.

#### National identity

For both life satisfaction and work motivation, the model (see Table 4b) suggested a significant sequential indirect effect via national identity via both social connectedness and game‐related socialising (ind_result_nationalid_socialconn_lifesat_: *B* = 0.01, CI_95%_ [0.005, 0.02]; ind_result_nationalid_gameresol_lifesat_: *B* = 0.01, CI_95%_ [0.003, 0.01]; ind_result_nationalid_socialconn_workmotiv_: *B* = 0.01, CI_95%_ [0.005, 0.02]; ind_result_nationalid_gameresol_workmotiv_: *B* = 0.01, CI_95%_ [0.004, 0.02]).

**TABLE 4B:** Summary of sequential indirect effects of game result (Win vs. Loss): National identity.

Outcome	Stage‐1 mediator	Stage‐2 mediator	*B*	CI_95%_	Interpretation
Life satisfaction	National identity	Social connectedness	0.01	[0.005, 0.02]	Significant
		Game‐related socialising	0.01	[0.003, 0.01]	Significant
Work motivation	National identity	Social connectedness	0.01	[0.005, 0.02]	Significant
		Game‐related socialising	0.01	[0.004, 0.02]	Significant

*Note*: Indirect effects are based on serial mediation analyses (PROCESS Model 6; Hayes, 2018), with Stage‐1 mediators (team, football, or national identity) and Stage‐2 mediators (social connectedness and game‐related socialising). Unstandardised coefficients (*B*) are reported. Confidence intervals (CI_95%_) are based on bootstrapping; effects are statistically significant when the confidence interval does not include zero.

### Interplay of social context and game result

Finally, we investigated the potential interplay between the social context (i.e., watching with others vs. alone) and game result (i.e., win vs. loss) on life satisfaction, work motivation and social identities (see Figure 1). We found no evidence of any interaction effects on life satisfaction, *B* = 0.31, *SE* = 0.20, *p* = .103, or on work motivation, *B* = −0.07, *SE* = 0.30, *p* = .816, nor for the social identities related to team identity, *B* = 0.06, *SE* = 0.21, *p* = .781, nor on football identity, *B* = −0.03, *SE* = 0.19, *p* = .862, and national identity, *B* = 0.25, *SE* = 0.20, *p* = .223.

In sum, when it comes to both work motivation and life satisfaction, the data point to the presence of two largely independent main effects associated with (1) watching the game with others (vs. alone) and (2) one's national team winning (vs. losing) the day before. Watching with others was associated with lower work motivation (via increased alcohol consumption) but watching one's team win coincided with higher work motivation and greater life satisfaction (via multiple levels of social identities and both social game‐related socialising and a sense of social connectedness).

## GENERAL DISCUSSION

Previous research has shown that both the social context (e.g., Neville & Reicher, 2011) and the sport itself (e.g., Unanue et al., 2022) have important psychological consequences. However, prior research has tended to look at these things in isolation and focused largely on well‐being outcomes. The present work investigates how both factors operate in tandem and examines their spill‐over effects into the work domain. Tracking participants over a four‐week period enabled us to compare the distinct and interactive relation of the social context of watching and the game result, as well as to assess the key drivers of these spill‐over effects.

### Theoretical implications

From a theoretical standpoint, this research was grounded in social identity theory (Tajfel & Turner, 1979) and self‐categorisation theory (Turner et al., 1994), both of which explain how individuals' cognitions, emotions, and behaviours are shaped by their identity with social groups and the sense of shared identity that flows from this (Haslam, 2004). By situating our findings within this framework, we conceptualise international sporting events as contexts in which group‐based identities increase and through which individual well‐being and motivation can rise or fall.

Surprisingly, watching games with others (vs. alone) was associated with reduced work motivation the following day. Although this had not been anticipated, we were able to gain more insight into this effect by looking at the role of alcohol consumption as a potential mechanism. Doing so suggested that lower motivation resulted from having consumed more alcohol on the previous day. From a social identity perspective, this finding suggests that while collective gatherings can strengthen a sense of belonging and social connection, referring to the ‘social cure’, they may also activate behavioural norms that are not always health‐promoting. This aligns with the ‘social curse’ framework (Haslam et al., 2018; Howell et al., 2014), which highlights that the same group processes that enhance well‐being can also impair individuals' functioning when group norms promote harmful behaviours.

Alongside, the social context of watching the game with others (vs. alone) did not predict higher life satisfaction. Aligned with this finding, shared viewing experiences were also not associated with increased social identity or heightened socialising. This contrasts with previous evidence that watching sporting games with others tends to foster a sense of collective identity (e.g., Neville & Reicher, 2011) but also speaks to the point that the identities that are activated by sporting events are highly context specific (Oakes et al., 1994). In particular, the absence of effects in the present study may reflect a mismatch between the social identities in the viewing context (i.e., defined by who participants watched the game with) and the types of social identities that were measured here. Specifically, it is possible that watching with friends or family activated concrete, interpersonal identities (e.g., as a friend, family member) rather than broader, abstract identities such as national identities. This is descriptively supported by our sample being biased towards respondents who watched the game at home with friends and family (*n* = 1514) rather than in large venues with strangers (e.g., public screening areas [*n* = 248] and stadiums [*n* = 2]; Neville & Reicher, 2011).

In contrast, there was broad support for the effects of game result. A win (vs. loss) was associated with stronger identification with football fans, with one's team, and with one's nation. These enhanced identities, in turn, were all associated with a sense of social connectedness and game‐related socialising, and ultimately with both life satisfaction and work motivation. This pattern aligns with and extends previous evidence that game results affect well‐being through team identity (Jang et al., 2018; Unanue et al., 2022) in showing that it can also bolster fan and national identity. However, the latter national identity effect was less pronounced in the full mediation model, suggesting that team and fan identities are more closely implicated in game result than national identities. Interestingly, our findings extend previous literature in showing a similar pattern for work motivation, suggesting spill‐over effects beyond the private domain and into the work domain.

From a theoretical standpoint, these findings provide nuanced insights into the operation of social identity processes in suggesting that different group‐based experiences have differential associations of both the extent of identification and key psychological outcomes. Specifically, abstract collective identities (e.g., football identity) appear to be more responsive to game results than they are to the social context in which those outcomes are observed. In part, this may reflect the way this is measured, but evidence of the effects of ingroup win clearly underlines the role of event‐relevant cues in activating multiple identities that spill over into daily life.

Interestingly, national identity emerged as a weaker mediator when tested alongside team and football identification. One explanation for this pattern is that different identity levels vary in their situational responsiveness and functional relevance. It seems plausible that proximal identities—such as identification with the team or with football—are closely tied to the immediate event context and thus more sensitive to state fluctuations in collective experiences such as game outcomes (Jang et al., 2018; Stieler & Germelmann, 2016). In contrast, national identity represents a more abstract and chronically accessible identity, with a presumably stronger trait component (see Fleeson, 2001, 2004); national identity may hence be less responsive to situational cues (Oakes et al., 1994; Turner et al., 1994). Accordingly, the present findings suggest that identity‐based spill‐over effects from sporting events are primarily driven by contextually grounded identities (i.e., team, football) rather than more abstract collective identities (i.e., country).

### Practical implications

Beyond its theoretical contributions, the present research also has practical implications that help to understand how large‐scale collective events coincide with everyday psychological functioning and experiences. At a practical level, our findings provide intriguing evidence that international sporting events are linked to employees' motivation—and that game results are particularly strongly linked to this outcome. The correlational mediation role of alcohol consumption in contributing to the demotivating association of watching games with others is also worth noting; it underlines the point that social group life can be beneficial as well as detrimental (e.g., Howell et al., 2014).

Our findings suggest that collective successes, such as an ingroup win, can have short‐term benefits that extend beyond the event itself, enhancing individuals' motivation in other life domains, including at work. At the same time, the results highlight that shared social experiences do not uniformly coincide with more positive outcomes. In particular, social contexts that are linked to behaviours such as alcohol consumption may go hand in hand with impaired functioning and energy levels the next day.

These insights are relevant for organisations and policymakers alike. For instance, employers may anticipate short‐term fluctuations in employees' motivation around major sporting events, while public health and event organisers may consider how to foster social engagement without reinforcing detrimental norms (e.g., excessive drinking). More broadly, the findings underscore that collective experiences can act as both a ‘social cure’ and a ‘social curse’, depending on the social dynamics they activate. This in turn highlights the importance of shaping group contexts in ways that promote beneficial rather than harmful spill‐over effects.

### Limitations and future research

Several limitations should be considered. First, the use of single‐item measures may limit reliability, induce measurement error, and inflate associations among conceptually related constructs. Future studies would benefit from using brief validated multi‐item measures when replicating the reported relationships. Second, our mediation analyses relied on same‐wave data, constraining causal inference. Future work could temporally separate mediators and outcome measures to gain more specific insights. Third, work motivation was operationalised as a rather broad, state‐level construct, without distinguishing between motivational qualities or clearly separating motivation from related constructs such as general activation or energy. In addition, responses may reflect variation in daily routines (e.g., weekdays vs. weekends; see SOM). In future studies it would be beneficial to gain more fine‐grained insights into different forms of motivation. Fourth, the social context variable (alone vs. with others) aggregates qualitatively distinct viewing settings, potentially obscuring important variation in identity processes, which future studies could address. Fifth, although the data span multiple countries, cross‐national differences were not explicitly modelled or sufficiently powered for (that being said, see SOM for country‐based robustness analyses). Finally, the self‐selected sample of football‐interested participants may limit generalisability. Both limitations could be addressed in future research.

## CONCLUSION

International sporting events like UEFA EURO 2024 take place globally. While previous research has focused largely on economic or societal outcomes, surprisingly little attention has been paid to psychological, individual‐level spill‐over effects on well‐being and work. Our study addresses this gap by examining how social context and game result contribute to next‐day changes in spectators' life satisfaction and work motivation. Our findings show that game results, particularly team victories, elevate social identification, social connectedness and game‐related socialising, which in turn predict positive outcomes of elevated life satisfaction and work motivation.

From a social identity theory perspective, these findings highlight that the psychological consequences of international sporting events are rooted in the way individuals identify as members of collective entities. When these identities are affirmed through collective success, they provide emotional and motivational resources, the ‘social cure’. When social participation encourages maladaptive norms (e.g., excessive drinking), the same identities can become a ‘social curse’ (Haslam et al., 2018).

In answering the question posed in the title, the emotional and psychological ripple effects of international sporting events appear to be linked more clearly by ‘whether we win’ than they are by ‘who we watch with’. This underscores the influential role of collective success in shaping well‐being and motivation—both at home and at work—and makes the point that the link of international sporting events is as much eudaimonic as it is economic.

## AUTHOR CONTRIBUTIONS

**Meikel Neumann:** Conceptualization; writing – original draft; methodology; writing – review and editing; formal analysis; visualization. **Annik Strauch:** Conceptualization; writing – original draft; methodology; writing – review and editing; formal analysis; visualization. **Lea Boecker:** Conceptualization. **Kathi Diel:** Conceptualization. **Yannik A. Escher:** Investigation; data curation; supervision. **Paweł Muniak:** Conceptualization. **Danna Oomen:** Conceptualization. **Hannes M. Petrowsky:** Investigation; data curation; supervision. **Leonie Schwarz:** Conceptualization. **Marcel Weber:** Conceptualization. **Malte Friese:** Conceptualization; resources; supervision. **Oliver Genschow:** Conceptualization; resources; funding acquisition. **S. Alexander Haslam:** Conceptualization; writing – review and editing; supervision. **David D. Loschelder:** Conceptualization; writing – review and editing; supervision; resources.

## CONFLICT OF INTEREST STATEMENT

The authors declare that they have no conflict of interest.

## Supporting information


**Table S1.** Three‐Level Social Context Variable (Alone vs. With Others at Home vs. With Others in Public): Frequency and Descriptives Statistics for All Variables.
**Table S2.** Three‐Level Social Context Variable (Alone vs. With Others at Home vs. With Others in Public): Intercorrelations for All Variables at Both Within– and Between‐Level.
**Table S3a.** England: Frequency and Descriptives Statistics for All Variables – Binary Social Context Variable.
**Table S3b.** England: Frequency and Descriptives Statistics for All Variables – Three–Categorical Social Context Variable.
**Table S4a.** England: Intercorrelations for All Variables at Both Within– and Between–Level – Binary Social Context Variable.
**Table S4b.** England: Intercorrelations for All Variables at Both Within‐ and Between‐Level – Three–Categorical Social Context Variable.
**Table S5a.** Germany: Frequency and Descriptives Statistics for All Variables – Binary Social Context Variable.
**Table S5b.** Germany: Frequency and Descriptives Statistics for All Variables – Three‐Level Social Context Variable.
**Table S6a.** Germany: Intercorrelations for All Variables at Both Within– and Between–Level – Binary Social Context Variable.
**Table S6b.** Germany: Intercorrelations for All Variables at Both Within– and Between–Level – Three‐Level Social Context Variable.
**Table S7a.** Italy: Frequency and Descriptives Statistics for All Variables – Binary Social Context Variable.
**Table S7b.** Italy: Frequency and Descriptives Statistics for All Variables – Three‐Level Social Context Variable.
**Table S8a.** Italy: Intercorrelations for All Variables at Both Within‐ and Between‐Level – Binary Social Context Variable.
**Table S8b.** Italy: Intercorrelations for All Variables at Both Within‐ and Between‐Level – Three‐Level Social Context Variable.
**Table S9a.** Netherlands: Frequency and Descriptives Statistics for All Variables – Binary Social Context Variable.
**Table S9b.** Netherlands: Frequency and Descriptives Statistics for All Variables – Three‐Level Social Context Variable.
**Table S10a.** Netherlands: Intercorrelations for All Variables at Both Within‐ and Between‐Level – Binary Social Context Variable.
**Table S10b.** Netherlands: Intercorrelations for All Variables at Both Within‐ and Between‐Level – Three‐Level Social Context Variable.
**Table S11a.** Poland: Frequency and Descriptives Statistics for All Variables – Binary Social Context Variable.
**Table S11b.** Poland: Frequency and Descriptives Statistics for All Variables – Three‐Level Social Context Variable.
**Table S12a.** Poland: Intercorrelations for All Variables at Both Within‐ and Between‐Level – Binary Social Context Variable.
**Table S12b.** Poland: Intercorrelations for All Variables at Both Within‐ and Between‐Level – Three‐Level Social Context Variable.
**Table S13.** Results of Main, Sensitivity, and Exploratory Analysis for More Detailed Review of Binary vs. Three‐Level Social Context Variable.
**Table S14**. Results of Main and Generalizability Analysis Across Countries and Comparison Results.
**Table S15**. Results of Hypotheses Tests When One Country is Removed from the Dataset.
**Table S16**. Results of Main and Robustness Analysis for Detailed Review of Game Result Variable Including All Games vs. Only Games Followed by a Weekday

## Data Availability

Preregistration, analysis code, and supplementary materials are available on OSF–SOM /OSF–Pre–Registration. The data that support the findings of this study are from a larger research project (Petrowsky* et al., 2026). The data are openly available on OSF.

## Footnotes

^1^
To test whether the reported effects differ across countries, we specified RI‐ and RS‐models with country added as a Level‐3 variable. The higher‐level models improved the model fit for the vast majority of analyses, thus suggesting that country can account for systematic variation of spectators' responses. More importantly, however, the direction, magnitude and significance of all effects, and thus all conclusions, remained unchanged (see SOM Table S14 for an overview). Moreover, in the Level‐3 RS models, random slope variances at individual and country levels were consistently estimated as near zero. These exploratory moderation analyses suggest that the various associations investigated in the paper do not vary meaningfully across countries.

^2^
Please find descriptives and intercorrelations per country and additional analyses with a three‐categorical social context variable (0 = alone, 1 = with others at home, 2 = with others in public) and per country in the SOM.

^3^
A robustness analysis showed that the reported findings remained largely unchanged when we removed the games played on a Friday and Saturday—given that spectators from the recruited countries would not very likely work on the next day (i.e., Saturday, Sunday; see SOM Table S16). While the direct effect of social viewing context on work motivation was no longer significant, the indirect effect via alcohol consumption remained statistically significant and directionally consistent with the main analyses.

^4^
Model indices for the effects of social context on alcohol consumption: RI model *B* = 0.86, *SE* = 0.08, *t*(1,595) = 8.16, *p* < .001, RS model *B* = 0.92, *SE* = 0.11, *t*(274.25) = 8.13, *p* < .001. Model fit statistics supported the RS model: *χ* ^2^(2) = 54.27, *p* < .001, AIC_RS_ = 9,419.80 vs. AIC_RI_ = 9,470.10; BIC_RS_ = 9,454.60 vs. BIC_RI_ = 9,493.20. Model indices for the effects of alcohol consumption on work motivation: RI model *B* = −0.16, *SE* = 0.02, *t*(1,615.44) = −6.43, *p* < .001, RS model *B* = −0.16, SE = 0.03, *t*(204.52) = −5.82, *p* < .001. Model fit statistics supported the RI model: *χ* ^2^(2) = 3.64, *p* = .162, AIC_RI_ = 9,191.40 vs. AIC_RS_ = 9,191.80; BIC_RI_ = 9,214.60 vs. BIC_RS_ = 9,226.50.
