# Supplementary material for: Whether we win or with whom we watch: How international sporting events link to life satisfaction and work motivation
Source: Br J Soc Psychol. 2026 Aug 7;65(4):e70115. doi: 10.1111/bjso.70115 (PMC13451784; doi:10.1111/bjso.70115)
Supplement: Supplementary file 1 [file BJSO-65-0-s001.docx]

**Whether We Win or With Whom We Watch:**

**How International Sporting Events Link to Life Satisfaction and Work Motivation**

**Supplementary Online Material
(SOM)**

**Table of Content**

**Descriptives and Intercorrelations for Three-Level Social Context Variable and on Country-Level Analysis**

| **Table** | **Title** |
| --- | --- |
| **Table S1** | Three-Level Social Context Variable (Alone vs. With Others at Home vs. With Others in Public): Frequency and Descriptives Statistics for All Variables |
| **Table S2** | Three-Level Social Context Variable (Alone vs. With Others at Home vs. With Others in Public): Intercorrelations for All Variables at Both Within– and Between-Level |
| **Table S3a** | England: Frequency and Descriptives Statistics for All Variables – Binary Social Context Variable |
| **Table S3b** | England: Frequency and Descriptives Statistics for All Variables – Three–Categorical Social Context Variable |
| **Table S4a** | England: Intercorrelations for All Variables at Both Within– and Between–Level – Binary Social Context Variable |
| **Table S4b** | England: Intercorrelations for All Variables at Both Within- and Between-Level – Three–Categorical Social Context Variable |
| **Table S5a** | Germany: Frequency and Descriptives Statistics for All Variables – Binary Social Context Variable |
| **Table S5b** | Germany: Frequency and Descriptives Statistics for All Variables – Three-Level Social Context Variable |
| **Table S6a** | Germany: Intercorrelations for All Variables at Both Within– and Between–Level – Binary Social Context Variable |
| **Table S6b** | Germany: Intercorrelations for All Variables at Both Within– and Between–Level – Three-Level Social Context Variable |
| **Table S7a** | Italy: Frequency and Descriptives Statistics for All Variables – Binary Social Context Variable |
| **Table S7b** | Italy: Frequency and Descriptives Statistics for All Variables – Three-Level Social Context Variable |
| **Table S8a** | Italy: Intercorrelations for All Variables at Both Within- and Between-Level – Binary Social Context Variable |
| **Table S8b** | Italy: Intercorrelations for All Variables at Both Within- and Between-Level – Three-Level Social Context Variable |
| **Table S9a** | Netherlands: Frequency and Descriptives Statistics for All Variables – Binary Social Context Variable |
| **Table S9b** | Netherlands: Frequency and Descriptives Statistics for All Variables – Three-Level Social Context Variable |
| **Table S10a** | Netherlands: Intercorrelations for All Variables at Both Within- and Between-Level – Binary Social Context Variable |
| **Table S10b** | Netherlands: Intercorrelations for All Variables at Both Within- and Between-Level – Three-Level Social Context Variable |
| **Table S11a** | Poland: Frequency and Descriptives Statistics for All Variables – Binary Social Context Variable |
| **Table S11b** | Poland: Frequency and Descriptives Statistics for All Variables – Three-Level Social Context Variable |
| **Table S12a** | Poland: Intercorrelations for All Variables at Both Within- and Between-Level – Binary Social Context Variable |
| **Table S12b** | Poland: Intercorrelations for All Variables at Both Within- and Between-Level – Three-Level Social Context Variable |

**Additional Analyses for the Social Context Variable:**

| **Table S13** | Results of Main, Sensitivity, and Exploratory Analysis for More Detailed Review of Binary vs. Three-Level Social Context Variable |
| --- | --- |

**Additional Analyses Accounting for Country Variable:**

| **Table S14** | Results of Main and Generalizability Analysis Across Countries and Comparison Results |
| --- | --- |
| **Table S15** | Results of Hypotheses Tests When One Country is Removed from the Dataset |

**Additional Analyses for Game Result Variable:**

| **Table S16** | Results of Main and Robustness Analysis for Detailed Review of Game Result Variable Including All Games vs. Only Games Followed by a Weekday |
| --- | --- |

**Table S1**

*Three-Level Social Context Variable (Alone vs. With Others at Home vs. With Others in Public): Frequency and Descriptives Statistics for All Variables*

| **Variable** | **Overall** | **Game Result** | | **Social Context** | | |  |  |  |
| --- | --- | --- | --- | --- | --- | --- | --- | --- | --- |
|  |  | **Loss** | **Win** | **Alone** | **With Others At Home** | **With Others in Public** |  |  |  |
|  | **N (%)** | **N (%)** | **N (%)** | **N (%)** | **N (%)** | **N (%)** |  |  |  |
| **1. Game Result** |  |  |  |  |  |  |  |  |  |
| **Loss = 0** | 946 (39.2%) | — | — | 261 (40.2%), | 595 (39.3%), | 90 (36.0%), |  |  |  |
| **Win = 1** | 1,468 (60.8%) | — | — | 389 (59.8%) | 919 (60.7%) | 160 (64.0%) |  |  |  |
| **2. Social Context** |  |  |  |  |  |  |  |  |  |
| **Alone = 0** | 650 (26.9%) | 261 (27.6%) | 389 (26.5%) | — | — | — |  |  |  |
| **With Others at  Home = 1** | 1,514 (62.7%) | 595 (62.9%) | 919 (62.6%) | — | — | — |  |  |  |
| **With Others in  Public = 2** | 250 (10.4%) | 90 (9.5%) | 160 (10.9%) | — | — | — |  |  |  |
|  | **M (SD)** | **M (SD)** | **M (SD)** | **M (SD)** | **M (SD)** | **M (SD)** | **SD_person_** | **SD_games_** | **ICC** |
| **3. Team Identification** | 4.92 (1.40) | 4.56 (1.56) | 5.15 (1.24) | 4.87 (1.47) | 4.93 (1.38) | 4.99 (1.40) | 1.10 | 0.92 | .59 |
| **4. Football Identification** | 4.96 (1.47) | 4.75 (1.57) | 5.10 (1.38) | 4.91 (1.54) | 4.98 (1.44) | 5.02 (1.42) | 1.27 | 0.83 | .70 |
| **5. National Identification** | 4.41 (1.60) | 4.39 (1.62) | 4.42 (1.59) | 4.33 (1.67) | 4.42 (1.58) | 4.50 (1.49) | 1.36 | 0.87 | .71 |
| **6. Social Connectedness** | 4.47 (1.38) | 4.26 (1.43) | 4.60 (1.33) | 4.24 (1.46) | 4.55 (1.32) | 4.58 (1.48) | 0.97 | 1.00 | .48 |
| **7. Match-Related Socializing** | 4.65 (1.68) | 4.14 (1.79) | 4.97 (1.51) | 4.51 (1.81) | 4.66 (1.61) | 4.96 (1.70) | 1.01 | 1.35 | .36 |
| **8. Life  Satisfaction** | 4.82 (1.39) | 4.58 (1.40) | 4.97 (1.35) | 4.55 (1.46) | 4.91 (1.35) | 4.99 (1.30) | 1.10 | 0.84 | .63 |
| **9. Work Motivation** | 4.02 (1.77) | 3.83 (1.73) | 4.13 (1.80) | 4.06 (1.75) | 4.05 (1.77) | 3.69 (1.86) | 1.13 | 1.37 | .41 |
| **10. Alcohol Consumption** | 1.37 (1.97) | 1.26 (1.89) | 1.44 (2.02) | 0.75 (1.44) | 1.31 (1.89) | 3.36 (2.36) | 1.40 | 1.39 | .50 |

*Note. k* = 2,414 observations from 842 participants. *M (SD)* = mean and standard deviation; *n* (%) = frequency and percentage for binary variables. *SD_person_* = between-person SD; *SD_games_* = within-person SD (null multilevel models). ICC = intraclass correlation coefficient. Dashes (–) indicate not applicable values.

**Table S2**

*Three-Level Social Context Variable (Alone vs. With Others at Home vs. With Others in Public): Intercorrelations for All Variables at Both Within- and Between-Level*

| **Variable** | **1.** | **2.** | **3.** | **4.** | **5.** | **6.** | **7.** | **8.** | **9.** | **10.** |
| --- | --- | --- | --- | --- | --- | --- | --- | --- | --- | --- |
| **1. Game Result** |  | .10** | .12*** | .01 | -.08* | .02 | .05 | .08* | .02 | .14*** |
| **2. Social Context** | -.02 |  | .05 | .02 | .07* | .12*** | .14*** | .17*** | .01 | .34*** |
| **3. Team Identification** | .34*** | .01 |  | .73*** | .54*** | .39*** | .47*** | .25*** | .27*** | .04 |
| **4. Football Identification** | .26*** | .00 | .53*** |  | .41*** | .39*** | .51*** | .25*** | .22*** | .01 |
| **5. National Identification** | .10*** | -.03 | .34*** | .28*** |  | .41*** | .34*** | .28*** | .32*** | .02 |
| **6. Social Connectedness** | .19*** | .03 | .29*** | .24*** | .20*** |  | .45*** | .58*** | .44*** | -.06 |
| **7. Match-Related Socializing** | .38*** | .00 | .34*** | .35*** | .23*** | .29*** |  | .34*** | .27*** | .01 |
| **8. Life Satisfaction** | .24*** | -.01 | .26*** | .26*** | .20*** | .37*** | .29*** |  | .41*** | -.01 |
| **9. Work Motivation** | .13*** | -.10*** | .21*** | .22*** | .13*** | .25*** | .20*** | .28*** |  | -.13*** |
| **10. Alcohol Use** | .01 | .30*** | .01 | -.01 | .00 | .02 | -.01 | -.00 | -.16*** |  |

*Note.* Intercorrelations are displayed based on *k* = 2,414 observations from *N* = 842 participants. Below the diagonal are correlations at the game level (within-level). Above the diagonal are correlations at the person level (between-level). Game result is binary (loss = 0, win = 1), social context is three-level (alone = 0, with others at home = 1, with others in public = 2). Statistically significant bivariate correlations are displayed with asterisks. * *p <* .05, ** *p <* .01, *** *p <* .001.

**Table S3a**

*England: Frequency and Descriptives Statistics for All Variables – Binary Social Context Variable*

| **Variable** | **Overall** | **Game Result** | | **Social Context** | |  |  |  |
| --- | --- | --- | --- | --- | --- | --- | --- | --- |
|  |  | **Loss** | **Win** | **Alone** | **With Others** |  |  |  |
|  | **N (%)** | **N (%)** | **N (%)** | **N (%)** | **N (%)** |  |  |  |
| **1. Game Result** | 738 (100.0%) | — | — | 189 (100.0%) | 549 (100.0%) | — | — | — |
| Loss = 0 | 156 (21.1%) | — | — | 30 (15.9%) | 126 (23.0%) | — | — | — |
| Win = 1 | 582 (78.9%) | — | — | 159 (84.1%) | 423 (77.0%) | — | — | — |
| **2. Social Context** | 738 (100.0%) | 156 (100.0%) | 582 (100.0%) | — | — | — | — | — |
| Alone = 0 | 189 (25.6%) | 30 (19.2%) | 159 (27.3%) | — | — | — | — | — |
| With Others = 1 | 549 (74.4%) | 126 (80.8%) | 423 (72.7%) | — | — | — | — | — |
|  | **M (SD)** | **M (SD)** | **M (SD)** | **M (SD)** | **M (SD)** | **SD_person_** | **SD_games_** | **ICC** |
| **3. Team Identification** | 5.06 (1.32) | 4.63 (1.46) | 5.17 (1.26) | 5.15 (1.27) | 5.02 (1.34) | 1.01 | 0.86 | .58 |
| **4. Football Identification** | 5.24 (1.29) | 4.91 (1.48) | 5.33 (1.23) | 5.31 (1.21) | 5.22 (1.32) | 1.07 | 0.77 | .65 |
| **5. National Identification** | 4.48 (1.56) | 4.37 (1.57) | 4.51 (1.56) | 4.52 (1.58) | 4.47 (1.56) | 1.33 | 0.84 | .72 |
| **6. Social Connectedness** | 4.67 (1.30) | 4.31 (1.41) | 4.77 (1.26) | 4.63 (1.29) | 4.69 (1.31) | 0.90 | 0.95 | .47 |
| **7. Match-Related Socializing** | 4.92 (1.57) | 4.16 (1.63) | 5.12 (1.50) | 5.04 (1.65) | 4.87 (1.55) | 0.95 | 1.27 | .36 |
| **8. Life Satisfaction** | 4.88 (1.42) | 4.48 (1.51) | 4.99 (1.37) | 4.85 (1.37) | 4.89 (1.44) | 1.19 | 0.80 | .69 |
| **9. Work Motivation** | 4.38 (1.71) | 3.96 (1.79) | 4.50 (1.66) | 4.50 (1.58) | 4.34 (1.75) | 1.20 | 1.21 | .49 |
| **10. Alcohol Consumption** | 1.47 (2.09) | 1.68 (2.27) | 1.42 (2.03) | 1.07 (1.71) | 1.61 (2.19) | 1.50 | 1.44 | .52 |

*Note.* The English sample includes *k* = 738 observations from 182 participants. Game result: 0 = loss, 1 = win. Social context: 0 = alone, 1 = with others. *M (SD)* = mean and standard deviation; *n* (%) = frequency and percentage for binary variables. *SD_person_* = between-person SD; *SD_games_* = within-person SD (null multilevel models). ICC = intraclass correlation coefficient. Dashes (–) indicate not applicable values.

**Table S3b**

*England: Frequency and Descriptives Statistics for All Variables – Three-Level Social Context Variable*

| **Variable** | **Overall** | **Game Result** | | **Social Context** | | |  |  |  |
| --- | --- | --- | --- | --- | --- | --- | --- | --- | --- |
|  |  | **Loss** | **Win** | **Alone** | **With Others At Home** | **With Others in Public** |  |  |  |
|  | **N (%)** | **N (%)** | **N (%)** | **N (%)** | **N (%)** | **N (%)** |  |  |  |
| **1. Game Result** |  |  |  |  |  |  |  |  |  |
| **Loss = 0** | 156 (21.1%), | — | — | 30 (15.9%), | 114 (23.3%), | 12 (20.3%), |  |  |  |
| **Win = 1** | 582 (78.9%) | — | — | 159 (84.1%) | 376 (76.7%) | 47 (79.7%) |  |  |  |
| **2. Social Context** |  |  |  |  |  |  |  |  |  |
| **Alone = 0** | 189 (25.6%), | 30 (19.2%), | 159 (27.3%), | — | — | — |  |  |  |
| **With Others at  Home = 1** | 490 (66.4%), | 114 (73.1%), | 376 (64.6%), | — | — | — |  |  |  |
| **With Others in  Public = 2** | 59 (8.0%) | 12 (7.7%) | 47 (8.1%) | — | — | — |  |  |  |
|  | **M (SD)** | **M (SD)** | **M (SD)** | **M (SD)** | **M (SD)** | **M (SD)** | **SD_person_** | **SD_games_** | **ICC** |
| **3. Team Identification** | 5.06 (1.32) | 4.63 (1.46) | 5.17 (1.26) | 5.15 (1.27) | 5.00 (1.35) | 5.19 (1.18) | 1.01 | 0.86 | .58 |
| **4. Football Identification** | 5.24 (1.29) | 4.91 (1.48) | 5.33 (1.23) | 5.31 (1.21) | 5.18 (1.34) | 5.49 (1.10) | 1.07 | 0.77 | .65 |
| **5. National Identification** | 4.48 (1.56) | 4.37 (1.57) | 4.51 (1.56) | 4.52 (1.58) | 4.46 (1.55) | 4.53 (1.60) | 1.33 | 0.84 | .72 |
| **6. Social Connectedness** | 4.67 (1.30) | 4.31 (1.41) | 4.77 (1.26) | 4.63 (1.29) | 4.65 (1.31) | 4.98 (1.27) | 0.90 | 0.95 | .47 |
| **7. Match-Related Socializing** | 4.92 (1.57) | 4.16 (1.63) | 5.12 (1.50) | 5.04 (1.65) | 4.81 (1.54) | 5.37 (1.52) | 0.95 | 1.27 | .36 |
| **8. Life  Satisfaction** | 4.88 (1.42) | 4.48 (1.51) | 4.99 (1.37) | 4.85 (1.37) | 4.86 (1.45) | 5.14 (1.29) | 1.19 | 0.80 | .69 |
| **9. Work Motivation** | 4.38 (1.71) | 3.96 (1.79) | 4.50 (1.66) | 4.50 (1.58) | 4.38 (1.71) | 4.07 (2.03) | 1.20 | 1.21 | .49 |
| **10. Alcohol Consumption** | 1.47 (2.09) | 1.68 (2.27) | 1.42 (2.03) | 1.07 (1.71) | 1.35 (1.99) | 3.81 (2.49) | 1.50 | 1.44 | .52 |

*Note.* The English sample includes *k* = 738 observations from 182 participants. *M (SD)* = mean and standard deviation; *n* (%) = frequency and percentage for binary variables. *SD_person_* = between-person SD; *SD_games_* = within-person SD (null multilevel models). ICC = intraclass correlation coefficient. Dashes (–) indicate not applicable values.

**Table S4a**

*England: Intercorrelations for All Variables at Both Within- and Between-Level – Binary Social Context Variable*

| **Variable** | **1.** | **2.** | **3.** | **4.** | **5.** | **6.** | **7.** | **8.** | **9.** | **10.** |
| --- | --- | --- | --- | --- | --- | --- | --- | --- | --- | --- |
| **1. Game Result (win = 1)** |  | -.11 | .05 | .00 | -.01 | -.01 | .03 | .09 | .08 | .03 |
| **2. Social Context (with others = 1)** | -.12** |  | -.01 | .01 | .01 | .08 | .07 | .10 | .04 | .06 |
| **3. Team Identification** | .30*** | -.03 |  | .70*** | .68*** | .47*** | .59*** | .41*** | .41*** | -.02 |
| **4. Football Identification** | .26*** | -.04 | .57*** |  | .45*** | .52*** | .58*** | .41*** | .29*** | -.00 |
| **5. National Identification** | .08* | -.03 | .38*** | .39*** |  | .34*** | .42*** | .33*** | .45*** | .01 |
| **6. Social Connectedness** | .23*** | -.01 | .34*** | .27*** | .27*** |  | .55*** | .66*** | .47*** | -.15* |
| **7. Match-Related Socializing** | .36*** | -.07* | .41*** | .37*** | .25*** | .35*** |  | .41*** | .38*** | -.08 |
| **8. Life Satisfaction** | .27*** | -.05 | .34*** | .34*** | .25*** | .38*** | .34*** |  | .55*** | -.08 |
| **9. Work Motivation** | .20*** | -.08* | .23*** | .25*** | .16*** | .26*** | .25*** | .30*** |  | -.28*** |
| **10. Alcohol Consumption** | -.09* | .14*** | -.06 | -.00 | -.04 | .05 | -.02 | -.01 | -.16*** |  |

*Note.* Intercorrelations are displayed based on *k* = 738 observations from *n* = 182 participants. Below the diagonal are correlations at the game level (within-level). Above the diagonal are correlations at the person level (between-level). Statistically significant bivariate correlations are displayed with asterisks. * *p <* .05, ** *p <* .01, *** *p <* .001.

**Table S4b**

*England: Intercorrelations for All Variables at Both Within- and Between-Level – Three-Level Social Context Variable*

| **Variable** | **1.** | **2.** | **3.** | **4.** | **5.** | **6.** | **7.** | **8.** | **9.** | **10.** |
| --- | --- | --- | --- | --- | --- | --- | --- | --- | --- | --- |
| **1. Game Result** |  | -.05 | .05 | .00 | -.01 | -.01 | .03 | .09 | .08 | .03 |
| **2. Social Context** | -.09* |  | .04 | .07 | .03 | .12 | .14 | .13 | .03 | .18* |
| **3. Team Identification** | .30*** | -.03 |  | .70*** | .68*** | .47*** | .59*** | .41*** | .41*** | -.02 |
| **4. Football Identification** | .26*** | -.03 | .57*** |  | .45*** | .52*** | .58*** | .41*** | .29*** | -.00 |
| **5. National Identification** | .08* | -.01 | .38*** | .39*** |  | .34*** | .42*** | .33*** | .45*** | .01 |
| **6. Social Connectedness** | .23*** | .02 | .34*** | .27*** | .27*** |  | .55*** | .66*** | .47*** | -.15* |
| **7. Match-Related Socializing** | .36*** | -.04 | .41*** | .37*** | .25*** | .35*** |  | .41*** | .38*** | -.08 |
| **8. Life Satisfaction** | .27*** | -.03 | .34*** | .34*** | .25*** | .38*** | .34*** |  | .55*** | -.08 |
| **9. Work Motivation** | .20*** | -.10** | .23*** | .25*** | .16*** | .26*** | .25*** | .30*** |  | -.28*** |
| **10. Alcohol Use** | -.09* | .31*** | -.06 | -.00 | -.04 | .05 | -.02 | -.01 | -.16*** |  |

*Note.* Intercorrelations are displayed based on *k* = 738 observations from *n* = 182 participants. Below the diagonal are correlations at the game level (within-level). Above the diagonal are correlations at the person level (between-level). Game result is binary (loss = 0, win = 1), social context is three-level (alone = 0, with others at home = 1, with others in public = 2). Statistically significant bivariate correlations are displayed with asterisks. * *p <* .05, ** *p <* .01, *** *p <* .001.

**Table S5a**

*Germany: Frequency and Descriptives Statistics for All Variables – Binary Social Context Variable*

| **Variable** | **Overall** | **Game Result** | | **Social Context** | |  |  |  |
| --- | --- | --- | --- | --- | --- | --- | --- | --- |
|  |  | **Loss** | **Win** | **Alone** | **With Others** |  |  |  |
|  | **N (%)** | **N (%)** | **N (%)** | **N (%)** | **N (%)** |  |  |  |
| **1. Game Result** | 447 (100.0%) | — | — | 142 (100.0%) | 305 (100.0%) | — | — | — |
| Loss = 0 | 104 (23.3%) | — | — | 31 (21.8%) | 73 (23.9%) | — | — | — |
| Win = 1 | 343 (76.7%) | — | — | 111 (78.2%) | 232 (76.1%) | — | — | — |
| **2. Social Context** | 447 (100.0%) | 104 (100.0%) | 343 (100.0%) | — | — | — | — | — |
| Alone = 0 | 142 (31.8%) | 31 (29.8%) | 111 (32.4%) | — | — | — | — | — |
| With Others = 1 | 305 (68.2%) | 73 (70.2%) | 232 (67.6%) | — | — | — | — | — |
|  | **M (SD)** | **M (SD)** | **M (SD)** | **M (SD)** | **M (SD)** | **SD_person_** | **SD_games_** | **ICC** |
| **3. Team Identification** | 4.90 (1.43) | 4.65 (1.67) | 4.98 (1.35) | 4.82 (1.59) | 4.94 (1.35) | 1.16 | 0.87 | .64 |
| **4. Football Identification** | 4.85 (1.55) | 4.65 (1.64) | 4.91 (1.52) | 4.70 (1.80) | 4.92 (1.42) | 1.39 | 0.76 | .77 |
| **5. National Identification** | 4.01 (1.76) | 3.90 (1.74) | 4.05 (1.76) | 3.93 (1.81) | 4.05 (1.73) | 1.54 | 0.89 | .75 |
| **6. Social Connectedness** | 4.25 (1.48) | 4.00 (1.49) | 4.33 (1.47) | 3.98 (1.61) | 4.38 (1.40) | 1.04 | 1.05 | .49 |
| **7. Match-Related Socializing** | 4.76 (1.62) | 4.35 (1.85) | 4.89 (1.53) | 4.40 (1.83) | 4.93 (1.49) | 1.06 | 1.21 | .43 |
| **8. Life Satisfaction** | 4.64 (1.46) | 4.31 (1.41) | 4.74 (1.47) | 4.32 (1.52) | 4.79 (1.41) | 1.09 | 0.95 | .57 |
| **9. Work Motivation** | 3.52 (1.81) | 3.12 (1.73) | 3.64 (1.82) | 3.65 (1.85) | 3.46 (1.79) | 1.09 | 1.44 | .36 |
| **10. Alcohol Consumption** | 1.45 (2.04) | 1.33 (2.04) | 1.49 (2.04) | 0.65 (1.37) | 1.83 (2.19) | 1.54 | 1.35 | .56 |

*Note.* The German sample includes *k* = 447 observations from *n* = 170 participants. Game result: 0 = loss, 1 = win. Social context: 0 = alone, 1 = with others. *M (SD)* = mean and standard deviation; *n* (%) = frequency and percentage for binary variables. *SD_person_* = between-person SD; *SD_games_* = within-person SD (null multilevel models). ICC = intraclass correlation coefficient. Dashes (–) indicate not applicable values.

**Table S5b**

*Germany: Frequency and Descriptives Statistics for All Variables – Three-Level Social Context Variable*

| **Variable** | **Overall** | **Game Result** | | **Social Context** | | |  |  |  |
| --- | --- | --- | --- | --- | --- | --- | --- | --- | --- |
|  |  | **Loss** | **Win** | **Alone** | **With Others At Home** | **With Others in Public** |  |  |  |
|  | **N (%)** | **N (%)** | **N (%)** | **N (%)** | **N (%)** | **N (%)** |  |  |  |
| **1. Game Result** |  |  |  |  |  |  |  |  |  |
| **Loss = 0** | 104 (23.3%), | — | — | 31 (21.8%), | 60 (25.0%), | 13 (20.0%), |  |  |  |
| **Win = 1** | 343 (76.7%) | — | — | 111 (78.2%) | 180 (75.0%) | 52 (80.0%) |  |  |  |
| **2. Social Context** |  |  |  |  |  |  |  |  |  |
| **Alone = 0** | 142 (31.8%), | 31 (29.8%), | 111 (32.4%), | — | — | — |  |  |  |
| **With Others at  Home = 1** | 240 (53.7%), | 60 (57.7%), | 180 (52.5%), | — | — | — |  |  |  |
| **With Others in  Public = 2** | 65 (14.5%) | 13 (12.5%) | 52 (15.2%) | — | — | — |  |  |  |
|  | **M (SD)** | **M (SD)** | **M (SD)** | **M (SD)** | **M (SD)** | **M (SD)** | **SD_person_** | **SD_games_** | **ICC** |
| **3. Team Identification** | 4.90 (1.43) | 4.65 (1.67) | 4.98 (1.35) | 4.82 (1.59) | 4.87 (1.37) | 5.20 (1.25) | 1.16 | 0.87 | .64 |
| **4. Football Identification** | 4.85 (1.55) | 4.65 (1.64) | 4.91 (1.52) | 4.70 (1.80) | 4.90 (1.45) | 5.00 (1.32) | 1.39 | 0.76 | .77 |
| **5. National Identification** | 4.01 (1.76) | 3.90 (1.74) | 4.05 (1.76) | 3.93 (1.81) | 3.90 (1.74) | 4.60 (1.60) | 1.54 | 0.89 | .75 |
| **6. Social Connectedness** | 4.25 (1.48) | 4.00 (1.49) | 4.33 (1.47) | 3.98 (1.61) | 4.30 (1.34) | 4.68 (1.58) | 1.04 | 1.05 | .49 |
| **7. Match-Related Socializing** | 4.76 (1.62) | 4.35 (1.85) | 4.89 (1.53) | 4.40 (1.83) | 4.82 (1.49) | 5.32 (1.40) | 1.06 | 1.21 | .43 |
| **8. Life  Satisfaction** | 4.64 (1.46) | 4.31 (1.41) | 4.74 (1.47) | 4.32 (1.52) | 4.71 (1.42) | 5.09 (1.37) | 1.09 | 0.95 | .57 |
| **9. Work Motivation** | 3.52 (1.81) | 3.12 (1.73) | 3.64 (1.82) | 3.65 (1.85) | 3.43 (1.75) | 3.55 (1.91) | 1.09 | 1.44 | .36 |
| **10. Alcohol Consumption** | 1.45 (2.04) | 1.33 (2.04) | 1.49 (2.04) | 0.65 (1.37) | 1.47 (2.02) | 3.14 (2.29) | 1.54 | 1.35 | .56 |

*Note.* The German sample includes *k* = 447 observations from *n* = 170 participants. *M (SD)* = mean and standard deviation; *n* (%) = frequency and percentage for binary variables. *SD_person_* = between-person SD; *SD_games_* = within-person SD (null multilevel models). ICC = intraclass correlation coefficient. Dashes (–) indicate not applicable values.

**Table S6a**

*Germany: Intercorrelations for All Variables at Both Within- and Between-Level – Binary Social Context Variable*

| **Variable** | **1.** | **2.** | **3.** | **4.** | **5.** | **6.** | **7.** | **8.** | **9.** | **10.** |
| --- | --- | --- | --- | --- | --- | --- | --- | --- | --- | --- |
| **1. Game Result (win = 1)** |  | .18* | -.01 | -.04 | .07 | -.01 | -.01 | .04 | .02 | .11 |
| **2. Social Context (with others = 1)** | -.13** |  | .06 | .08 | .07 | .23** | .28*** | .19* | .07 | .22** |
| **3. Team Identification** | .23*** | .06 |  | .73*** | .53*** | .46*** | .49*** | .33*** | .26*** | .05 |
| **4. Football Identification** | .23*** | .04 | .56*** |  | .38*** | .32*** | .52*** | .28*** | .18* | -.02 |
| **5. National Identification** | .06 | -.05 | .23*** | .13** |  | .50*** | .29*** | .37*** | .37*** | .05 |
| **6. Social Connectedness** | .20*** | .01 | .23*** | .20*** | .08 |  | .51*** | .63*** | .38*** | .00 |
| **7. Match-Related Socializing** | .28*** | -.03 | .15** | .20*** | .18*** | .32*** |  | .37*** | .22** | .06 |
| **8. Life Satisfaction** | .26*** | -.01 | .18*** | .16*** | .09 | .49*** | .32*** |  | .33*** | .00 |
| **9. Work Motivation** | .21*** | -.15** | .16*** | .15** | .11* | .33*** | .11* | .30*** |  | -.13 |
| **10. Alcohol Consumption** | -.00 | .30*** | .10* | .01 | .08 | -.06 | .03 | -.04 | -.23*** |  |

*Note.* Intercorrelations are displayed based on *k* = 447 observations from *n* = 170 participants. Below the diagonal are correlations at the game level (within-level). Above the diagonal are correlations at the person level (between-level). Statistically significant bivariate correlations are displayed with asterisks. * *p <* .05, ** *p <* .01, *** *p <* .001.

**Table S6b**

*Germany: Intercorrelations for All Variables at Both Within- and Between-Level – Three–Categorical Social Context Variable*

| **Variable** | **1.** | **2.** | **3.** | **4.** | **5.** | **6.** | **7.** | **8.** | **9.** | **10.** |
| --- | --- | --- | --- | --- | --- | --- | --- | --- | --- | --- |
| **1. Game Result** |  | .23** | -.01 | -.04 | .07 | -.01 | -.01 | .04 | .02 | .11 |
| **2. Social Context** | -.09 |  | .08 | .06 | .14 | .24** | .28*** | .22** | .05 | .36*** |
| **3. Team Identification** | .23*** | .07 |  | .73*** | .53*** | .46*** | .49*** | .33*** | .26*** | .05 |
| **4. Football Identification** | .23*** | .02 | .56*** |  | .38*** | .32*** | .52*** | .28*** | .18* | -.02 |
| **5. National Identification** | .06 | -.06 | .23*** | .13** |  | .50*** | .29*** | .37*** | .37*** | .05 |
| **6. Social Connectedness** | .20*** | .02 | .23*** | .20*** | .08 |  | .51*** | .63*** | .38*** | .00 |
| **7. Match-Related Socializing** | .28*** | .01 | .15** | .20*** | .18*** | .32*** |  | .37*** | .22** | .06 |
| **8. Life Satisfaction** | .26*** | -.00 | .18*** | .16*** | .09 | .49*** | .32*** |  | .33*** | .00 |
| **9. Work Motivation** | .21*** | -.12* | .16*** | .15** | .11* | .33*** | .11* | .30*** |  | -.13 |
| **10. Alcohol Use** | -.00 | .32*** | .10* | .01 | .08 | -.06 | .03 | -.04 | -.23*** |  |

*Note.* Intercorrelations are displayed based on *k* = 447 observations from *n* = 170 participants. Below the diagonal are correlations at the game level (within-level). Above the diagonal are correlations at the person level (between-level). Game result is binary (loss = 0, win = 1), social context is three-level (alone = 0, with others at home = 1, with others in public = 2). Statistically significant bivariate correlations are displayed with asterisks. * *p <* .05, ** *p <* .01, *** *p <* .001.

**Table S7a**

*Italy: Frequency and Descriptives Statistics for All Variables – Binary Social Context Variable*

| **Variable** | **Overall** | **Game Result** | | **Social Context** | |  |  |  |
| --- | --- | --- | --- | --- | --- | --- | --- | --- |
|  |  | **Loss** | **Win** | **Alone** | **With Others** |  |  |  |
|  | **N (%)** | **N (%)** | **N (%)** | **N (%)** | **N (%)** |  |  |  |
| **1. Game Result** | 338 (100.0%) | — | — | 87 (100.0%) | 251 (100.0%) | — | — | — |
| Loss = 0 | 228 (67.5%) | — | — | 63 (72.4%) | 165 (65.7%) | — | — | — |
| Win = 1 | 110 (32.5%) | — | — | 24 (27.6%) | 86 (34.3%) | — | — | — |
| **2. Social Context** | 338 (100.0%) | 228 (100.0%) | 110 (100.0%) | — | — | — | — | — |
| Alone = 0 | 87 (25.7%) | 63 (27.6%) | 24 (21.8%) | — | — | — | — | — |
| With Others = 1 | 251 (74.3%) | 165 (72.4%) | 86 (78.2%) | — | — | — | — | — |
|  | **M (SD)** | **M (SD)** | **M (SD)** | **M (SD)** | **M (SD)** | **SD_person_** | **SD_games_** | **ICC** |
| **3. Team Identification** | 4.67 (1.56) | 4.41 (1.63) | 5.22 (1.23) | 4.53 (1.55) | 4.72 (1.56) | 1.09 | 1.09 | .50 |
| **4. Football Identification** | 4.72 (1.67) | 4.63 (1.66) | 4.91 (1.68) | 4.66 (1.72) | 4.74 (1.65) | 1.42 | 0.89 | .72 |
| **5. National Identification** | 4.27 (1.57) | 4.16 (1.57) | 4.49 (1.56) | 4.26 (1.63) | 4.27 (1.55) | 1.23 | 0.93 | .64 |
| **6. Social Connectedness** | 4.09 (1.57) | 4.03 (1.57) | 4.21 (1.57) | 3.76 (1.51) | 4.20 (1.58) | 1.02 | 1.19 | .43 |
| **7. Match-Related Socializing** | 3.93 (1.86) | 3.72 (1.83) | 4.35 (1.85) | 3.54 (1.95) | 4.06 (1.81) | 1.17 | 1.43 | .40 |
| **8. Life Satisfaction** | 4.47 (1.38) | 4.39 (1.39) | 4.64 (1.35) | 4.26 (1.32) | 4.54 (1.39) | 1.02 | 0.91 | .56 |
| **9. Work Motivation** | 3.73 (1.78) | 3.71 (1.75) | 3.76 (1.84) | 3.75 (1.69) | 3.73 (1.81) | 1.17 | 1.33 | .44 |
| **10. Alcohol Consumption** | 1.56 (1.86) | 1.51 (1.84) | 1.67 (1.92) | 0.78 (1.43) | 1.83 (1.92) | 1.16 | 1.47 | .38 |

*Note.* The Italian sample includes *k* = 338 observations from 159 participants. Game result: 0 = loss, 1 = win. Social context: 0 = alone, 1 = with others. *M (SD)* = mean and standard deviation; *n* (%) = frequency and percentage for binary variables. *SD_person_* = between-person SD; *SD_games_* = within-person SD (null multilevel models). ICC = intraclass correlation coefficient. Dashes (–) indicate not applicable values.

**Table S7b**

*Italy: Frequency and Descriptives Statistics for All Variables – Three-Level Social Context Variable*

| **Variable** | **Overall** | **Game Result** | | **Social Context** | | |  |  |  |
| --- | --- | --- | --- | --- | --- | --- | --- | --- | --- |
|  |  | **Loss** | **Win** | **Alone** | **With Others At Home** | **With Others in Public** |  |  |  |
|  | **N (%)** | **N (%)** | **N (%)** | **N (%)** | **N (%)** | **N (%)** |  |  |  |
| **1. Game Result** |  |  |  |  |  |  |  |  |  |
| **Loss = 0** | 228 (67.5%), | — | — | 63 (72.4%), | 138 (67.3%), | 27 (58.7%), |  |  |  |
| **Win = 1** | 110 (32.5%) | — | — | 24 (27.6%) | 67 (32.7%) | 19 (41.3%) |  |  |  |
| **2. Social Context** |  |  |  |  |  |  |  |  |  |
| **Alone = 0** | 87 (25.7%), | 63 (27.6%), | 24 (21.8%), | — | — | — |  |  |  |
| **With Others at  Home = 1** | 205 (60.7%), | 138 (60.5%), | 67 (60.9%), | — | — | — |  |  |  |
| **With Others in  Public = 2** | 46 (13.6%) | 27 (11.8%) | 19 (17.3%) | — | — | — |  |  |  |
|  | **M (SD)** | **M (SD)** | **M (SD)** | **M (SD)** | **M (SD)** | **M (SD)** | **SD_person_** | **SD_games_** | **ICC** |
| **3. Team Identification** | 4.67 (1.56) | 4.41 (1.63) | 5.22 (1.23) | 4.53 (1.55) | 4.82 (1.52) | 4.28 (1.66) | 1.09 | 1.09 | .50 |
| **4. Football Identification** | 4.72 (1.67) | 4.63 (1.66) | 4.91 (1.68) | 4.66 (1.72) | 4.85 (1.62) | 4.26 (1.72) | 1.42 | 0.89 | .72 |
| **5. National Identification** | 4.27 (1.57) | 4.16 (1.57) | 4.49 (1.56) | 4.26 (1.63) | 4.31 (1.53) | 4.07 (1.64) | 1.23 | 0.93 | .64 |
| **6. Social Connectedness** | 4.09 (1.57) | 4.03 (1.57) | 4.21 (1.57) | 3.76 (1.51) | 4.26 (1.53) | 3.93 (1.76) | 1.02 | 1.19 | .43 |
| **7. Match-Related Socializing** | 3.93 (1.86) | 3.72 (1.83) | 4.35 (1.85) | 3.54 (1.95) | 4.06 (1.82) | 4.07 (1.78) | 1.17 | 1.43 | .40 |
| **8. Life  Satisfaction** | 4.47 (1.38) | 4.39 (1.39) | 4.64 (1.35) | 4.26 (1.32) | 4.60 (1.41) | 4.30 (1.31) | 1.02 | 0.91 | .56 |
| **9. Work Motivation** | 3.73 (1.78) | 3.71 (1.75) | 3.76 (1.84) | 3.75 (1.69) | 3.83 (1.80) | 3.26 (1.79) | 1.17 | 1.33 | .44 |
| **10. Alcohol Consumption** | 1.56 (1.86) | 1.51 (1.84) | 1.67 (1.92) | 0.78 (1.43) | 1.70 (1.82) | 2.41 (2.26) | 1.16 | 1.47 | .38 |

*Note.* The Italian sample includes *k* = 338 observations from 159 participants. *M (SD)* = mean and standard deviation; *n* (%) = frequency and percentage for binary variables. *SD_person_* = between-person SD; *SD_games_* = within-person SD (null multilevel models). ICC = intraclass correlation coefficient. Dashes (–) indicate not applicable values.

**Table S8a**

*Italy: Intercorrelations for All Variables at Both Within- and Between-Level – Binary Social Context Variable*

| **Variable** | **1.** | **2.** | **3.** | **4.** | **5.** | **6.** | **7.** | **8.** | **9.** | **10.** |
| --- | --- | --- | --- | --- | --- | --- | --- | --- | --- | --- |
| **1. Game Result (win = 1)** |  | .08 | .20* | .00 | .06 | -.23** | .11 | -.06 | -.09 | .10 |
| **2. Social Context (with others = 1)** | .09 |  | .11 | .03 | .04 | .09 | .14 | .13 | .01 | .30*** |
| **3. Team Identification** | .39*** | -.08 |  | .70*** | .62*** | .33*** | .38*** | .24** | .24** | -.01 |
| **4. Football Identification** | .17** | -.06 | .47*** |  | .47*** | .42*** | .46*** | .28*** | .26*** | -.03 |
| **5. National Identification** | .18*** | -.16** | .42*** | .25*** |  | .39*** | .37*** | .31*** | .29*** | -.04 |
| **6. Social Connectedness** | .25*** | .07 | .36*** | .17** | .25*** |  | .34*** | .60*** | .60*** | -.19* |
| **7. Match-Related Socializing** | .26*** | .01 | .31*** | .29*** | .29*** | .34*** |  | .31*** | .28*** | -.08 |
| **8. Life Satisfaction** | .22*** | -.12* | .30*** | .29*** | .35*** | .31*** | .28*** |  | .38*** | -.10 |
| **9. Work Motivation** | .09 | -.01 | .29*** | .28*** | .33*** | .37*** | .28*** | .27*** |  | -.17* |
| **10. Alcohol Consumption** | .06 | .10 | -.04 | -.08 | .03 | .05 | -.09 | .04 | -.04 |  |

*Note.* Intercorrelations are displayed based on *k* = 338 observations from *n* = 159 participants. Below the diagonal are correlations at the game level (within-level). Above the diagonal are correlations at the person level (between-level). Statistically significant bivariate correlations are displayed with asterisks. * *p <* .05, ** *p <* .01, *** *p <* .001.

**Table S8b**

*Italy: Intercorrelations for All Variables at Both Within- and Between-Level – Three–Categorical Social Context Variable*

| **Variable** | **1.** | **2.** | **3.** | **4.** | **5.** | **6.** | **7.** | **8.** | **9.** | **10.** |
| --- | --- | --- | --- | --- | --- | --- | --- | --- | --- | --- |
| **1. Game Result** |  | .14 | .20* | .00 | .06 | -.23** | .11 | -.06 | -.09 | .10 |
| **2. Social Context** | .11 |  | .02 | -.05 | -.01 | -.02 | .10 | .07 | -.07 | .40*** |
| **3. Team Identification** | .39*** | -.08 |  | .70*** | .62*** | .33*** | .38*** | .24** | .24** | -.01 |
| **4. Football Identification** | .17** | -.07 | .47*** |  | .47*** | .42*** | .46*** | .28*** | .26*** | -.03 |
| **5. National Identification** | .18*** | -.09 | .42*** | .25*** |  | .39*** | .37*** | .31*** | .29*** | -.04 |
| **6. Social Connectedness** | .25*** | .05 | .36*** | .17** | .25*** |  | .34*** | .60*** | .60*** | -.19* |
| **7. Match-Related Socializing** | .26*** | .03 | .31*** | .29*** | .29*** | .34*** |  | .31*** | .28*** | -.08 |
| **8. Life Satisfaction** | .22*** | -.14** | .30*** | .29*** | .35*** | .31*** | .28*** |  | .38*** | -.10 |
| **9. Work Motivation** | .09 | -.05 | .29*** | .28*** | .33*** | .37*** | .28*** | .27*** |  | -.17* |
| **10. Alcohol Use** | .06 | .07 | -.04 | -.08 | .03 | .05 | -.09 | .04 | -.04 |  |

*Note.* Intercorrelations are displayed based on *k* = 338 observations from *n* = 159 participants. Below the diagonal are correlations at the game level (within-level). Above the diagonal are correlations at the person level (between-level). Game result is binary (loss = 0, win = 1), social context is three-level (alone = 0, with others at home = 1, with others in public = 2). Statistically significant bivariate correlations are displayed with asterisks. * *p <* .05, ** *p <* .01, *** *p <* .001.

**Table S9a**

*Netherlands: Frequency and Descriptives Statistics for All Variables – Binary Social Context Variable*

| **Variable** | **Overall** | **Game Result** | | **Social Context** | |  |  |  |
| --- | --- | --- | --- | --- | --- | --- | --- | --- |
|  |  | **Loss** | **Win** | **Alone** | **With Others** |  |  |  |
|  | **N (%)** | **N (%)** | **N (%)** | **N (%)** | **N (%)** |  |  |  |
| **1. Game Result** | 650 (100.0%) | — | — | 155 (100.0%) | 495 (100.0%) | — | — | — |
| Loss = 0 | 217 (33.4%) | — | — | 60 (38.7%) | 157 (31.7%) | — | — | — |
| Win = 1 | 433 (66.6%) | — | — | 95 (61.3%) | 338 (68.3%) | — | — | — |
| **2. Social Context** | 650 (100.0%) | 217 (100.0%) | 433 (100.0%) | — | — | — | — | — |
| Alone = 0 | 155 (23.8%) | 60 (27.6%) | 95 (21.9%) | — | — | — | — | — |
| With Others = 1 | 495 (76.2%) | 157 (72.4%) | 338 (78.1%) | — | — | — | — | — |
|  | **M (SD)** | **M (SD)** | **M (SD)** | **M (SD)** | **M (SD)** | **SD_person_** | **SD_games_** | **ICC** |
| **3. Team Identification** | 4.98 (1.25) | 4.44 (1.31) | 5.24 (1.12) | 4.94 (1.40) | 4.99 (1.20) | 0.88 | 0.90 | .49 |
| **4. Football Identification** | 4.81 (1.43) | 4.43 (1.52) | 5.00 (1.34) | 4.73 (1.49) | 4.84 (1.41) | 1.11 | 0.93 | .58 |
| **5. National Identification** | 4.48 (1.43) | 4.29 (1.42) | 4.57 (1.44) | 4.48 (1.47) | 4.48 (1.42) | 1.11 | 0.88 | .61 |
| **6. Social Connectedness** | 4.59 (1.25) | 4.39 (1.33) | 4.69 (1.20) | 4.39 (1.27) | 4.66 (1.24) | 0.80 | 0.95 | .42 |
| **7. Match-Related Socializing** | 4.52 (1.63) | 3.54 (1.63) | 5.01 (1.40) | 4.48 (1.70) | 4.53 (1.61) | 0.58 | 1.52 | .13 |
| **8. Life Satisfaction** | 5.09 (1.24) | 4.84 (1.32) | 5.22 (1.18) | 4.72 (1.40) | 5.21 (1.16) | 0.95 | 0.82 | .57 |
| **9. Work Motivation** | 4.10 (1.80) | 4.04 (1.74) | 4.12 (1.83) | 4.32 (1.78) | 4.03 (1.80) | 0.90 | 1.56 | .25 |
| **10. Alcohol Consumption** | 1.26 (1.95) | 1.04 (1.79) | 1.37 (2.01) | 0.44 (1.07) | 1.52 (2.09) | 1.45 | 1.41 | .52 |

*Note.* The Dutch sample includes *k* = 650 observations from 181 participants. Game result: 0 = loss, 1 = win. Social context: 0 = alone, 1 = with others. *M (SD)* = mean and standard deviation; *n* (%) = frequency and percentage for binary variables. *SD_person_* = between-person SD; *SD_games_* = within-person SD (null multilevel models). ICC = intraclass correlation coefficient. Dashes (–) indicate not applicable values.

**Table S9b**

*Netherlands: Frequency and Descriptives Statistics for All Variables – Three-Level Social Context Variable*

| **Variable** | **Overall** | **Game Result** | | **Social Context** | | |  |  |  |
| --- | --- | --- | --- | --- | --- | --- | --- | --- | --- |
|  |  | **Loss** | **Win** | **Alone** | **With Others At Home** | **With Others in Public** |  |  |  |
|  | **N (%)** | **N (%)** | **N (%)** | **N (%)** | **N (%)** | **N (%)** |  |  |  |
| **1. Game Result** |  |  |  |  |  |  |  |  |  |
| **Loss = 0** | 217 (33.4%), | — | — | 60 (38.7%), | 131 (30.7%), | 26 (38.2%), |  |  |  |
| **Win = 1** | 433 (66.6%) | — | — | 95 (61.3%) | 296 (69.3%) | 42 (61.8%) |  |  |  |
| **2. Social Context** |  |  |  |  |  |  |  |  |  |
| **Alone = 0** | 155 (23.8%), | 60 (27.6%), | 95 (21.9%), | — | — | — |  |  |  |
| **With Others at  Home = 1** | 427 (65.7%), | 131 (60.4%), | 296 (68.4%), | — | — | — |  |  |  |
| **With Others in  Public = 2** | 68 (10.5%) | 26 (12.0%) | 42 (9.7%) | — | — | — |  |  |  |
|  | **M (SD)** | **M (SD)** | **M (SD)** | **M (SD)** | **M (SD)** | **M (SD)** | **SD_person_** | **SD_games_** | **ICC** |
| **3. Team Identification** | 4.98 (1.25) | 4.44 (1.31) | 5.24 (1.12) | 4.94 (1.40) | 4.95 (1.19) | 5.21 (1.20) | 0.88 | 0.90 | .49 |
| **4. Football Identification** | 4.81 (1.43) | 4.43 (1.52) | 5.00 (1.34) | 4.73 (1.49) | 4.81 (1.41) | 5.04 (1.35) | 1.11 | 0.93 | .58 |
| **5. National Identification** | 4.48 (1.43) | 4.29 (1.42) | 4.57 (1.44) | 4.48 (1.47) | 4.45 (1.47) | 4.63 (1.11) | 1.11 | 0.88 | .61 |
| **6. Social Connectedness** | 4.59 (1.25) | 4.39 (1.33) | 4.69 (1.20) | 4.39 (1.27) | 4.68 (1.24) | 4.50 (1.24) | 0.80 | 0.95 | .42 |
| **7. Match-Related Socializing** | 4.52 (1.63) | 3.54 (1.63) | 5.01 (1.40) | 4.48 (1.70) | 4.50 (1.58) | 4.72 (1.82) | 0.58 | 1.52 | .13 |
| **8. Life  Satisfaction** | 5.09 (1.24) | 4.84 (1.32) | 5.22 (1.18) | 4.72 (1.40) | 5.21 (1.16) | 5.22 (1.16) | 0.95 | 0.82 | .57 |
| **9. Work Motivation** | 4.10 (1.80) | 4.04 (1.74) | 4.12 (1.83) | 4.32 (1.78) | 4.05 (1.81) | 3.88 (1.73) | 0.90 | 1.56 | .25 |
| **10. Alcohol Consumption** | 1.26 (1.95) | 1.04 (1.79) | 1.37 (2.01) | 0.44 (1.07) | 1.14 (1.80) | 3.88 (2.22) | 1.45 | 1.41 | .52 |

*Note.* The Dutch sample includes *k* = 650 observations from 181 participants. *M (SD)* = mean and standard deviation; *n* (%) = frequency and percentage for binary variables. *SD_person_* = between-person SD; *SD_games_* = within-person SD (null multilevel models). ICC = intraclass correlation coefficient. Dashes (–) indicate not applicable values.

**Table S10a**

*Netherlands: Intercorrelations for All Variables at Both Within- and Between-Level – Binary Social Context Variable*

| **Variable** | **1.** | **2.** | **3.** | **4.** | **5.** | **6.** | **7.** | **8.** | **9.** | **10.** |
| --- | --- | --- | --- | --- | --- | --- | --- | --- | --- | --- |
| **1. Game Result (win = 1)** |  | .02 | .26*** | .14 | .15* | .15* | .23** | .13 | .02 | .08 |
| **2. Social Context (with others = 1)** | .09* |  | -.00 | .05 | .06 | .16* | .04 | .28*** | -.09 | .24** |
| **3. Team Identification** | .46*** | .05 |  | .76*** | .47*** | .36*** | .44*** | .19* | .16* | .15* |
| **4. Football Identification** | .33*** | .06 | .54*** |  | .35*** | .32*** | .45*** | .17* | .11 | .13 |
| **5. National Identification** | .12** | .01 | .34*** | .30*** |  | .38*** | .25*** | .22** | .15* | .20** |
| **6. Social Connectedness** | .14*** | .03 | .29*** | .30*** | .20*** |  | .44*** | .60*** | .29*** | .02 |
| **7. Match-Related Socializing** | .52*** | .02 | .41*** | .44*** | .24*** | .23*** |  | .27*** | .12 | .22** |
| **8. Life Satisfaction** | .23*** | .05 | .22*** | .26*** | .14*** | .33*** | .28*** |  | .33*** | .07 |
| **9. Work Motivation** | .04 | -.08* | .17*** | .21*** | .07 | .15*** | .17*** | .25*** |  | -.11 |
| **10. Alcohol Consumption** | .10* | .27*** | .08* | .03 | -.01 | .01 | .02 | .01 | -.17*** |  |

*Note.* Intercorrelations are displayed based on *k* = 650 observations from *N* = 181 participants. Below the diagonal are correlations at the game level (within-level). Above the diagonal are correlations at the person level (between-level). Statistically significant bivariate correlations are displayed with asterisks. * *p <* .05, ** *p <* .01, *** *p <* .001.

**Table S10b**

*Netherlands: Intercorrelations for All Variables at Both Within- and Between-Level – Three–Categorical Social Context Variable*

| **Variable** | **1.** | **2.** | **3.** | **4.** | **5.** | **6.** | **7.** | **8.** | **9.** | **10.** |
| --- | --- | --- | --- | --- | --- | --- | --- | --- | --- | --- |
| **1. Game Result** |  | -.04 | .26*** | .14 | .15* | .15* | .23** | .13 | .02 | .08 |
| **2. Social Context** | .04 |  | .04 | .08 | .12 | .08 | .11 | .25*** | -.04 | .45*** |
| **3. Team Identification** | .46*** | .05 |  | .76*** | .47*** | .36*** | .44*** | .19* | .16* | .15* |
| **4. Football Identification** | .33*** | .04 | .54*** |  | .35*** | .32*** | .45*** | .17* | .11 | .13 |
| **5. National Identification** | .12** | .00 | .34*** | .30*** |  | .38*** | .25*** | .22** | .15* | .20** |
| **6. Social Connectedness** | .14*** | .02 | .29*** | .30*** | .20*** |  | .44*** | .60*** | .29*** | .02 |
| **7. Match-Related Socializing** | .52*** | -.00 | .41*** | .44*** | .24*** | .23*** |  | .27*** | .12 | .22** |
| **8. Life Satisfaction** | .23*** | .01 | .22*** | .26*** | .14*** | .33*** | .28*** |  | .33*** | .07 |
| **9. Work Motivation** | .04 | -.11** | .17*** | .21*** | .07 | .15*** | .17*** | .25*** |  | -.11 |
| **10. Alcohol Use** | .10* | .41*** | .08* | .03 | -.01 | .01 | .02 | .01 | -.17*** |  |

*Note.* Intercorrelations are displayed based on *k* = 650 observations from *N* = 181 participants. Below the diagonal are correlations at the game level (within-level). Above the diagonal are correlations at the person level (between-level). Game result is binary (loss = 0, win = 1), social context is three-level (alone = 0, with others at home = 1, with others in public = 2). Statistically significant bivariate correlations are displayed with asterisks. * *p <* .05, ** *p <* .01, *** *p <* .001.

**Table S11a**

*Poland: Frequency and Descriptives Statistics for All Variables – Binary Social Context Variable*

| **Variable** | **Overall** | **Game Result** | | **Social Context** | |  |  |  |
| --- | --- | --- | --- | --- | --- | --- | --- | --- |
|  |  | **Loss** | **Win** | **Alone** | **With Others** |  |  |  |
|  | **N (%)** | **N (%)** | **N (%)** | **N (%)** | **N (%)** |  |  |  |
| **1. Game Result** | 241 (100.0%) | — | — | 77 (100.0%) | 164 (100.0%) | — | — | — |
| Loss = 0 | 241 (100.0%) | — | — | 77 (100.0%) | 164 (100.0%) | — | — | — |
| Win = 1 | 0 (0.0%) | — | — | 0 (0.0%) | 0 (0.0%) | — | — | — |
| **2. Social Context** | 241 (100.0%) | 241 (100.0%) | 0 (NaN%) | — | — | — | — | — |
| Alone = 0 | 77 (32.0%) | 77 (32.0%) | 0 (NaN%) | — | — | — | — | — |
| With Others = 1 | 164 (68.0%) | 164 (68.0%) | 0 (NaN%) | — | — | — | — | — |
|  | **M (SD)** | **M (SD)** | **M (SD)** | **M (SD)** | **M (SD)** | **SD_person_** | **SD_games_** | **ICC** |
| **3. Team Identification** | 4.73 (1.69) | 4.73 (1.69) | NA | 4.47 (1.62) | 4.85 (1.72) | 1.31 | 1.08 | .60 |
| **4. Football Identification** | 5.08 (1.50) | 5.08 (1.50) | NA | 4.97 (1.50) | 5.13 (1.50) | 1.36 | 0.65 | .82 |
| **5. National Identification** | 4.92 (1.69) | 4.92 (1.69) | NA | 4.40 (1.97) | 5.16 (1.48) | 1.51 | 0.71 | .82 |
| **6. Social Connectedness** | 4.44 (1.35) | 4.44 (1.35) | NA | 4.03 (1.56) | 4.63 (1.19) | 0.97 | 0.93 | .52 |
| **7. Match-Related Socializing** | 4.97 (1.65) | 4.97 (1.65) | NA | 4.56 (1.79) | 5.16 (1.54) | 1.24 | 1.06 | .58 |
| **8. Life Satisfaction** | 4.72 (1.37) | 4.72 (1.37) | NA | 4.26 (1.64) | 4.94 (1.17) | 1.20 | 0.70 | .75 |
| **9. Work Motivation** | 3.99 (1.58) | 3.99 (1.58) | NA | 3.58 (1.64) | 4.18 (1.51) | 1.19 | 1.06 | .56 |
| **10. Alcohol Consumption** | 0.94 (1.59) | 0.94 (1.59) | NA | 0.75 (1.36) | 1.02 (1.68) | 1.21 | 0.98 | .60 |

*Note.* The Polish sample includes *k* = 241 observations from 150 participants. Game result: 0 = loss, 1 = win. Social context: 0 = alone, 1 = with others. *M (SD)* = mean and standard deviation; *n* (%) = frequency and percentage for binary variables. *SD_person_* = between-person SD; *SD_games_* = within-person SD (null multilevel models). ICC = intraclass correlation coefficient. The missing values (NA = not available) come from the polish team not winning any game during the tournament. Dashes (–) indicate not applicable values.

**Table S11b**

*Poland: Frequency and Descriptives Statistics for All Variables – Three-Level Social Context Variable*

| **Variable** | **Overall** | **Game Result** | | **Social Context** | | |  |  |  |
| --- | --- | --- | --- | --- | --- | --- | --- | --- | --- |
|  |  | **Loss** | **Win** | **Alone** | **With Others At Home** | **With Others in Public** |  |  |  |
|  | **N (%)** | **N (%)** | **N (%)** | **N (%)** | **N (%)** | **N (%)** |  |  |  |
| **1. Game Result** |  |  |  |  |  |  |  |  |  |
| **Loss = 0** | 241 (100%), | — | — | 77 (100%), | 152 (100%), | 12 (100%), |  |  |  |
| **Win = 1** | NA | — | — | NA | NA | NA |  |  |  |
| **2. Social Context** |  |  |  |  |  |  |  |  |  |
| **Alone = 0** | 77 (32.0%), | 77 (32.0%), | NA | — | — | — |  |  |  |
| **With Others at  Home = 1** | 152 (63.1%), | 152 (63.1%), | NA | — | — | — |  |  |  |
| **With Others in  Public = 2** | 12 (5.0%) | 12 (5.0%) | NA | — | — | — |  |  |  |
|  | **M (SD)** | **M (SD)** | **M (SD)** | **M (SD)** | **M (SD)** | **M (SD)** | **SD_person_** | **SD_games_** | **ICC** |
| **3. Team Identification** | 4.73 (1.69) | 4.73 (1.69) | NA | 4.47 (1.62) | 4.89 (1.69) | 4.33 (2.10) | 1.31 | 1.08 | .60 |
| **4. Football Identification** | 5.08 (1.50) | 5.08 (1.50) | NA | 4.97 (1.50) | 5.11 (1.51) | 5.50 (1.38) | 1.36 | 0.65 | .82 |
| **5. National Identification** | 4.92 (1.69) | 4.92 (1.69) | NA | 4.40 (1.97) | 5.19 (1.48) | 4.75 (1.54) | 1.51 | 0.71 | .82 |
| **6. Social Connectedness** | 4.44 (1.35) | 4.44 (1.35) | NA | 4.03 (1.56) | 4.60 (1.17) | 5.00 (1.41) | 0.97 | 0.93 | .52 |
| **7. Match-Related Socializing** | 4.97 (1.65) | 4.97 (1.65) | NA | 4.56 (1.79) | 5.12 (1.53) | 5.67 (1.67) | 1.24 | 1.06 | .58 |
| **8. Life  Satisfaction** | 4.72 (1.37) | 4.72 (1.37) | NA | 4.26 (1.64) | 4.93 (1.19) | 5.00 (0.85) | 1.20 | 0.70 | .75 |
| **9. Work Motivation** | 3.99 (1.58) | 3.99 (1.58) | NA | 3.58 (1.64) | 4.26 (1.49) | 3.17 (1.40) | 1.19 | 1.06 | .56 |
| **10. Alcohol Consumption** | 0.94 (1.59) | 0.94 (1.59) | NA | 0.75 (1.36) | 0.88 (1.54) | 2.92 (2.23) | 1.21 | 0.98 | .60 |

*Note.* The Polish sample includes *k* = 241 observations from 150 participants. *M (SD)* = mean and standard deviation; *n* (%) = frequency and percentage for binary variables. *SD_person_* = between-person SD; *SD_games_* = within-person SD (null multilevel models). ICC = intraclass correlation coefficient. The missing values (NA = not available) come from the polish team not winning any game during the tournament. Dashes (–) indicate not applicable values.

**Table S12a**

*Poland: Intercorrelations for All Variables at Both Within- and Between-Level – Binary Social Context Variable*

| **Variable** | **1.** | **2.** | **3.** | **4.** | **5.** | **6.** | **7.** | **8.** | **9.** | **10.** |
| --- | --- | --- | --- | --- | --- | --- | --- | --- | --- | --- |
| **1. Game Result (win = 1)** |  | NA | NA | NA | NA | NA | NA | NA | NA | NA |
| **2. Social Context (with others = 1)** | NA |  | .09 | .06 | .23** | .25** | .19* | .26** | .26** | .08 |
| **3. Team Identification** | NA | .11 |  | .77*** | .49*** | .33*** | .49*** | .07 | .22** | .03 |
| **4. Football Identification** | NA | .07 | .43*** |  | .38*** | .32*** | .51*** | .12 | .17* | .06 |
| **5. National Identification** | NA | .02 | .31*** | .07 |  | .40*** | .30*** | .17* | .24** | -.00 |
| **6. Social Connectedness** | NA | .05 | .04 | -.07 | -.05 |  | .39*** | .39*** | .34*** | .06 |
| **7. Match-Related Socializing** | NA | .22*** | .16* | .08 | -.03 | .16* |  | .29*** | .29*** | .11 |
| **8. Life Satisfaction** | NA | .21** | .18** | .20** | .30*** | .19** | .08 |  | .43*** | .04 |
| **9. Work Motivation** | NA | -.13 | .33*** | .28*** | .06 | .27*** | .23*** | .23*** |  | .07 |
| **10. Alcohol Consumption** | NA | .10 | -.12 | -.25*** | .08 | .05 | -.07 | .06 | -.11 |  |

*Note.* Intercorrelations are displayed based on *k* = 241 observations from *n* = 150 participants. Below the diagonal are correlations at the game level (within-level). Above the diagonal are correlations at the person level (between-level). Statistically significant bivariate correlations are displayed with asterisks. * *p <* .05, ** *p <* .01, *** *p <* .001. The missing values (NA = not available) come from the polish team not winning any game during the tournament.

**Table S12b**

*Poland: Intercorrelations for All Variables at Both Within- and Between-Level – Three-Level Social Context Variable*

| **Variable** | **1.** | **2.** | **3.** | **4.** | **5.** | **6.** | **7.** | **8.** | **9.** | **10.** |
| --- | --- | --- | --- | --- | --- | --- | --- | --- | --- | --- |
| **1. Game Result** |  | NA | NA | NA | NA | NA | NA | NA | NA | NA |
| **2. Social Context** | NA |  | .08 | .08 | .17* | .24** | .21** | .23** | .17* | .20* |
| **3. Team Identification** | NA | -.01 |  | .77*** | .49*** | .33*** | .49*** | .07 | .22** | .03 |
| **4. Football Identification** | NA | .07 | .43*** |  | .38*** | .32*** | .51*** | .12 | .17* | .06 |
| **5. National Identification** | NA | -.02 | .31*** | .07 |  | .40*** | .30*** | .17* | .24** | -.00 |
| **6. Social Connectedness** | NA | .09 | .04 | -.07 | -.05 |  | .39*** | .39*** | .34*** | .06 |
| **7. Match-Related Socializing** | NA | .22*** | .16* | .08 | -.03 | .16* |  | .29*** | .29*** | .11 |
| **8. Life Satisfaction** | NA | .20** | .18** | .20** | .30*** | .19** | .08 |  | .43*** | .04 |
| **9. Work Motivation** | NA | -.13 | .33*** | .28*** | .06 | .27*** | .23*** | .23*** |  | .07 |
| **10. Alcohol Use** | NA | .20** | -.12 | -.25*** | .08 | .05 | -.07 | .06 | -.11 |  |

*Note.* Intercorrelations are displayed based on *k* = 241 observations from *n* = 150 participants. Below the diagonal are correlations at the game level (within-level). Above the diagonal are correlations at the person level (between-level). Game result is binary (loss = 0, win = 1), social context is three-level (alone = 0, with others at home = 1, with others in public = 2). Statistically significant bivariate correlations are displayed with asterisks. * *p <* .05, ** *p <* .01, *** *p <* .001. The missing values (NA = not available) come from the Polish team not winning any game during the tournament.

**Table S13**

*Results of Main, Sensitivity, and Exploratory Analysis for More Detailed Review of Binary vs. Three-Level Social Context Variable*

| **Path** | **Main  (Alone vs Others)** | **Sensitivity  (Alone vs Home)** | **Exploratory  (Home vs Public)** |
| --- | --- | --- | --- |
| H1a: Social Context -> Life Satisfaction | RI; *b* = -0.005, *p* = .934 | RI; *b* = 0.030, *p* = .661 | RS; *b* = -0.060, *p* = .585 |
| H1b: Social Context -> Work Motivation | **RI; *b* = -0.382, *p <* .001** | **RI; *b* = -0.294, *p* = .010** | RI; *b* = -0.203, *p* = .215 |
| H2a: Social Context -> Team Identity | RI; *b* = 0.044, *p* = .538 | RI; *b* = 0.044, *p* = .562 | RI; *b* = 0.008, *p* = .943 |
| H2b: Social Context -> Football Identity | RI; *b* = 0.035, *p* = .581 | RI; *b* = 0.053, *p* = .443 | RI; *b* = -0.017, *p* = .859 |
| H2c: Social Context -> National Identity | RI; *b* = -0.091, *p* = .171 | RI; *b* = -0.142, *p* = .051 | RI; *b* = -0.063, *p* = .519 |
| H2d: Social Context -> Social Connectedness | RI; *b* = 0.062, *p* = .418 | RI; *b* = 0.038, *p* = .644 | RI; *b* = 0.020, *p* = .864 |
| H2e: Social Context -> Match-Rel. Socializing | RS; *b* = 0.033, *p* = .724 | RS; *b* = -0.055, *p* = 0.592 | RS; *b* = 0.139, *p* = .304 |
| H5a: Social Context x Result -> Life Satisfaction [social context] | RI; *b* = 0.000, *p* = .999 | RI; *b* = 0.045, *p* = .502 | RS; *b* = -0.061, *p* = .568 |
| H5a: Social Context x Result -> Life Satisfaction [result] | **RI; *b* = 0.418, *p <* .001** | **RI; *b* = 0.410, *p <* .001** | **RS; *b* = 0.461, *p <* .001** |
| H5a: Social Context x Result -> Life Satisfaction [interaction] | RI; *b* = 0.311, *p* = .103 | RI; *b* = 0.302, *p* = .139 | RS; *b* = 0.006, *p* = .984 |
| H5b: Social Context x Result -> Work Motivation [social context] | **RI; *b* = -0.370, *p <* .001** | **RI; *b* = -0.277, *p* = .014** | RI; *b* = -0.242, *p* = .141 |
| H5b: Social Context x Result -> Work Motivation [result] | **RI; *b* = 0.354, *p <* .001** | **RI; *b* = 0.316, *p <* .001** | **RI; *b* = 0.330, *p <* .001** |
| H5b: Social Context x Result -> Work Motivation [interaction] | RI; *b* = -0.071, *p* = .816 | RI; *b* = -0.054, *p* = .871 | RI; *b* = 0.796, *p* = .107 |
| H5: Social Context x Result -> Team Identity [social context] | RI; *b* = 0.061, *p* = .361 | RI; *b* = 0.074, *p* = .303 | RI; *b* = 0.032, *p* = .749 |
| H5: Social Context x Result -> Team Identity [result] | **RI; *b* = 0.643, *p <* .001** | **RI; *b* = 0.641, *p <* .001** | **RI; *b* = 0.632, *p <* .001** |
| H5: Social Context x Result -> Team Identity [interaction] | RI; *b* = 0.056, *p* = .782 | RI; *b* = 0.108, *p* = .621 | RI; *b* = -0.371, *p* = .250 |
| H5: Social Context x Result -> Football Identity [social context] | RI; *b* = 0.049, *p* = .426 | RI; *b* = 0.073, *p* = .269 | RI; *b* = -0.006, *p* = .952 |
| H5: Social Context x Result -> Football Identity [result] | **RI; *b* = 0.453, *p <* .001** | **RI; *b* = 0.461, *p <* .001** | **RI; *b* = 0.446, *p <* .001** |
| H5: Social Context x Result -> Football Identity [interaction] | RI; *b* = -0.033, *p* = .862 | RI; *b* = 0.129, *p* = .526 | RI; *b* = -0.148, *p* = .631 |
| H5: Social Context x Result -> National Identity [social context] | RI; *b* = -0.091, *p* = .169 | RI; *b* = -0.141, *p* = .052 | RI; *b* = -0.064, *p* = .522 |
| H5: Social Context x Result -> National Identity [result] | **RI; *b* = 0.178, *p <* .001** | **RI; *b* = 0.146, *p* = .003** | **RI; *b* = 0.216, *p <* .001** |
| H5: Social Context x Result -> National Identity [interaction] | RI; *b* = 0.247, *p* = .223 | RI; *b* = 0.344, *p* = .122 | RI; *b* = 0.036, *p* = .911 |
| EXPL: Social Context -> Alcohol Consumption | **RS; *b* = 0.917, *p <* .001** | **RS; *b* = 0.635, *p <* .001** | **RS; *b* = 1.147, *p <* .001** |
| EXPL: Alcohol Consumption -> Work Motivation | **RI; *b* = -0.157, *p <* .001** | **RI; *b* = -0.135, *p <* .001** | **RI; *b* = -0.164, *p <* .001** |

*Note.* All models including social context as a predictor are displayed. Significant results are displayed in bold. Significant results are displayed in bold. RI = Random-intercept model; RS = Random-intercept random-slope model. The better-fitting models are reported (RI vs. RS, based on χ² test, *p <* .05).

**Table S14**

*Results of Main Analysis (2-Level Models) and Generalizability Analysis Across Countries (3-Level-Models) and Comparison Results*

| **Path** | **2-Level Model** | **3-Level Model (+ Country)** | **Model Comparison 2-Level vs. 3-Level** |  |
| --- | --- | --- | --- | --- |
| ***H1: Social Context → Outcomes*** | | | | |
| H1a: Social Context → Life Satisfaction | RI; *b* = -0.005, *p* = .934 | RI; *b* = -0.005, *p* = .934 | **χ²(1) = 10.46, *p* = .001** |  |
| H1b: Social Context → Work Motivation | **RI; *b* = -0.382, *p <* .001** | **RI; *b* = -0.382, *p <* .001** | **χ²(1) = 26.38, *p <* .001** |  |
| ***H2: Social Context → Social Identities & Social Processes*** | | | | |
| H2a: Social Context → Team Identity | RI; *b* = 0.044, *p* = .538 | RI; *b* = 0.044, *p* = .538 | χ²(1) = 1.21, *p* = .272 |  |
| H2b: Social Context → Football Identity | RI; *b* = 0.035, *p* = .581 | RI; *b* = 0.035, *p* = .581 | **χ²(1) = 11.70, *p <* .001** |  |
| H2c: Social Context → National Identity | RI; *b* = -0.091, *p* = .171 | RI; *b* = -0.091, *p* = .171 | **χ²(1) = 20.07, *p <* .001** |  |
| H2d: Social Context → Social Connectedness | RI; *b* = 0.062, *p* = .418 | RI; *b* = 0.062, *p* = .418 | **χ²(1) = 15.03, *p <* .001** |  |
| H2e: Social Context → Match-Rel. Socializing | RS; *b* = 0.033, *p* = .724 | RS; *b* = 0.012, *p* = .891 | **χ²(3) = 182.12, *p <* .001** |  |
| ***H3: Social Identities → Social Processes*** | | | | |
| H3a: Team Identity → Social Connectedness | **RS; *b* = 0.303, *p <* .001** | **RS; *b* = 0.303, *p* = .043** | **χ²(3) = 15.20, *p* = .002** |  |
| H3b: Team Identity → Match-Rel. Socializing | **RS; *b* = 0.499, *p <* .001** | **RS; *b* = 0.437, *p* = .014** | **χ²(3) = 56.11, *p <* .001** |  |
| H3c: Football Identity → Social Connectedness | **RS; *b* = 0.268, *p <* .001** | **RS; *b* = 0.262, *p <* .001** | **χ²(3) = 15.22, *p* = .002** |  |
| H3d: Football Identity → Match-Rel. Socializing | **RS; *b* = 0.565, *p <* .001** | **RS; *b* = 0.509, *p* = .010** | **χ²(3) = 46.83, *p <* .001** |  |
| H3e: Country Identity → Social Connectedness | **RS; *b* = 0.201, *p <* .001** | RS; *b* = 0.190, *p* = .060 | **χ²(3) = 15.12, *p* = .002** |  |
| H3f: Country Identity → Match-Rel. Socializing | **RS; *b* = 0.352, *p <* .001** | **RS; *b* = 0.352, *p* = .003** | **χ²(3) = 46.14, *p <* .001** |  |
| ***H4: Social Processes → Outcomes*** | | | | |
| H4a: Social Connectedness → Life Satisfaction | **RS; *b* = 0.286, *p <* .001** | **RS; *b* = 0.280, *p* = .005** | **χ²(3) = 10.85, *p* = .013** |  |
| H4b: Social Connectedness → Work Motivation | **RS; *b* = 0.333, *p <* .001** | **RS; *b* = 0.349, *p* = 0.002** | **χ²(3) = 29.82, *p <* .001** |  |
| H4c: Match-Rel. Socializing → Life Satisfaction | **RS; *b* = 0.171, *p <* .001** | **RS; *b* = 0.173, *p <* .001** | **χ²(3) = 10.56, *p* = .014** |  |
| H4d: Match-Rel. Socializing → Work Motivation | **RI; *b* = 0.203, *p <* .001** | **RI; *b* = 0.203, *p <* .001** | **χ²(1) = 26.33, *p <* .001** |  |
| ***H5: Moderation — Social Context × Game Result*** | | | | |
| H5a: Soc. Context × Result → Life Satisfaction [social context] | RI; *b* = 0.000, *p* = .999 | RI; *b* = 0.000, *p* = .998 | **χ²(1) = 10.39, *p* = .001** |  |
| H5a: Soc. Context × Result → Life Satisfaction [result] | **RI; *b* = 0.418, *p <* .001** | **RI; *b* = 0.418, *p <* .001** | **χ²(1) = 10.39, *p* = .001** |  |
| H5a: Soc. Context × Result → Life Satisfaction [interaction] | RI; *b* = 0.311, *p* = .103 | RI; *b* = 0.307, *p* = .108 | **χ²(1) = 10.39, *p* = .001** |  |
| H5b: Soc. Context × Result → Work Motivation [social context] | **RI; *b* = -0.370, *p <* .001** | **RI; *b* = -0.370, *p <* .001** | **χ²(1) = 26.36, *p <* .001** |  |
| H5b: Soc. Context × Result → Work Motivation [result] | **RI; *b* = 0.354, *p <* .001** | **RI; *b* = 0.354, *p <* .001** | **χ²(1) = 26.36, *p <* .001** |  |
| H5b: Soc. Context × Result → Work Motivation [interaction] | RI; *b* = -0.071, *p* = .816 | RI; *b* = -0.071, *p* = .817 | **χ²(1) = 26.36, *p <* .001** |  |
| H5c: Soc. Context × Result → Team Identity [social context] | RI; *b* = 0.061, *p* = .361 | RI; *b* = 0.061, *p* = .362 | χ²(1) = 1.16, *p* = .281 |  |
| H5c: Soc. Context × Result → Team Identity [result] | **RI; *b* = 0.643, *p* < .001** | **RI; *b* = 0.642, *p* < .001** | χ²(1) = 1.16, *p* = .281 |  |
| H5c: Soc. Context × Result → Team Identity [interaction] | RI; *b* = 0.056, *p* = .782 | RI; *b* = 0.062, *p* = .759 | χ²(1) = 1.16, *p* = .281 |  |
| H5d: Soc. Context × Result → Football Identity [social context] | RI; *b* = 0.049, *p* = .426 | RI; *b* = 0.049, *p* = .430 | **χ²(1) = 11.74, *p* < .001** |  |
| H5d: Soc. Context × Result → Football Identity [result] | **RI; *b* = 0.453, *p* < .001** | **RI; *b* = 0.453, *p* < .001** | **χ²(1) = 11.74, *p* < .001** |  |
| H5d: Soc. Context × Result → Football Identity [interaction] | RI; *b* = -0.033, *p* = .862 | RI; *b* = -0.016, *p* = .932 | **χ²(1) = 11.74, *p* < .001** |  |
| H5e: Soc. Context × Result → National Identity [social context] | RI; *b* = -0.091, *p* = .169 | RI; *b* = -0.091, *p* = .170 | **χ²(1) = 19.96, *p* < .001** |  |
| H5e: Soc. Context × Result → National Identity [result] | **RI; *b* = 0.178, *p* < .001** | **RI; *b* = 0.178, *p* < .001** | **χ²(1) = 19.96, *p* < .001** |  |
| H5e: Soc. Context × Result → National Identity [interaction] | RI; *b* = 0.247, *p* = .223 | RI; *b* = 0.235, *p* = .246 | **χ²(1) = 19.96, *p* < .001** |  |
| ***EXPL: Alcohol Consumption as Mediator*** | | | | |
| EXPL: Social Context → Alcohol Consumption | **RS; *b* = 0.917, *p <* .001** | **RS; *b* = 0.845, *p* = 0.021** | χ²(3) = 5.34, *p* = .149 |  |
| EXPL: Alcohol Consumption → Work Motivation | **RI; *b* = -0.157, *p <* .001** | **RI; *b* = -0.157, *p <* .001** | **χ²(1) = 26.36, *p <* .001** |  |
| ***EXPL: Game Result → Outcomes, Identities & Social Processes*** | | | | |
| EXPL: Game Result → Life Satisfaction | **RS; *b* = 0.427, *p <* .001** | **RS; *b* = 0.426, *p <* .001** | **χ²(3) = 10.44, *p* = .015** |  |
| EXPL: Game Result → Work Motivation | **RI; *b* = 0.358, *p <* .001** | **RI; *b* = 0.358, *p <* .001** | **χ²(1) = 26.37, *p <* .001** |  |
| EXPL: Game Result → Team Identity | **RS; *b* = 0.662, *p <* .001** | **RS; *b* = 0.658, *p* = .006** | χ²(3) = 3.55, *p* = .314 |  |
| EXPL: Game Result → Football Identity | **RS; *b* = 0.465, *p <* .001** | **RS; *b* = 0.454, *p* = .007** | **χ²(3) = 12.54, *p* = 0.006** |  |
| EXPL: Game Result → National Identity | **RS; *b* = 0.184, *p <* .001** | **RS; *b* = 0.187, *p* = .002** | **χ²(3) = 20.33, *p <* .001** |  |
| EXPL: Game Result → Social Connectedness | **RS; *b* = 0.406, *p <* .001** | **RS; *b* = 0.413, *p* = .005** | **χ²(3) = 15.05, *p* = .002** |  |
| EXPL: Game Result → Match-Rel. Socializing | **RS; *b* = 1.028, *p <* .001** | **RS; *b* = 0.992, *p* = .016** | **χ²(3) = 54.31, *p <* .001** |  |
| **Number of models with same direction** | **41 out of 41 models** | |  |  |
| **Number of models with similar significance** | **40 out of 41 models** | |  |  |

*Note*. Significant results are displayed in bold. RI = Random-intercept model; RS = Random-intercept random-slope model. The better-fitting 2-level model (RI vs. RS, based on χ² test, *p <* .05) is reported alongside the corresponding 3-level model (adding country as level 3).

**Table S15**

*Results of Hypotheses Tests When One Country is Removed from the Dataset*

| **Path** | **Full Sample** | **Without England** | **Without Germany** | **Without Italy** | **Without Netherlands** | **Without Poland** |
| --- | --- | --- | --- | --- | --- | --- |
| H1a: Social Context → Life Sat. | RI; *b =* -0.005, *p =* .934 | RI; *b =* 0.046, *p =* .557 | RI; *b =* 0.000, *p =* .999 | RI; *b =* 0.032, *p =* .632 | RI; *b =* -0.073, *p =* .362 | RI; *b =* -0.038, *p =* .578 |
| H1b: Social Context → Work Motiv. | **RI; *b =* -0.382, *p <* .001** | **RI; *b =* -0.397, *p =* .003** | **RI; *b =* -0.320, *p =* .006** | **RI; *b =* -0.419, *p <* .001** | **RI; *b =* -0.385, *p =* .001** | **RI; *b =* -0.385, *p <* .001** |
| H2a: Social Context → Team ID | RI; *b =* 0.044, *p =* .538 | RI; *b =* 0.094, *p =* .278 | RI; *b =* 0.019, *p =* .809 | RI; *b =* 0.078, *p =* .284 | RI; *b =* 0.003, *p =* .973 | RI; *b =* 0.022, *p =* .766 |
| H2b: Social Context → Football ID | RI; *b =* 0.035, *p =* .581 | RI; *b =* 0.089, *p =* .257 | RI; *b =* 0.024, *p =* .738 | RI; *b =* 0.057, *p =* .387 | RI; *b =* -0.030, *p =* .686 | RI; *b =* 0.029, *p =*.671 |
| H2c: Social Context → National ID | RI; *b =* -0.091, *p =* .171 | RI; *b =* -0.096, *p =* .234 | RI; *b =* -0.081, *p =* .273 | RI; *b =* -0.046, *p =* .503 | RI; *b =* -0.145, *p =* .074 | RI; *b =* -0.102, *p =* .145 |
| Social Context → Soc. Connect. | RI; *b =* 0.062, *p =* .418 | RI; *b =* 0.102, *p =* .276 | RI; *b =* 0.068, *p =* .421 | RI; *b =* 0.042, *p =*.596 | RI; *b =* 0.047, *p =* .624 | RI; *b =* 0.058, *p =* .471 |
| Social Context → Match-Rel. Soc. | RS; *b =* 0.033, *p =* .724 | RS; *b =* 0.098, *p =* .389 | RS; *b =* 0.032, *p =* .761 | RS; *b =* 0.022, *p =* .816 | RS; *b =* 0.049, *p =* .655 | RS; *b =* -0.025, *p =* .799 |
| H3: Team ID → Soc. Connect. | **RS; *b =* 0.303, *p <* .001** | **RS; *b =* 0.285, *p <* .001** | **RS; *b =* 0.308, *p <* .001** | **RS; *b =* 0.280, *p <* .001** | **RS; *b =* 0.312, *p <* .001** | **RS; *b =* 0.327, *p <* .001** |
| H3: Team ID → Match-Rel. Soc. | **RS; *b =* 0.499, *p <* .001** | **RI; *b =* 0.456, *p <* .001** | **RS; *b =* 0.558, *p <* .001** | **RS; *b =* 0.509, *p <* .001** | **RS; *b =* 0.430, *p <* .001** | **RS; *b =* 0.529, *p <* .001** |
| H3: Football ID → Soc. Connect. | **RS; *b =* 0.268, *p <* .001** | **RS; *b =* 0.249, *p <* .001** | **RS; *b =* 0.268, *p <* .001** | **RS; *b =* 0.268, *p <* .001** | **RS; *b =* 0.265, *p <* .001** | **RS; *b =* 0.285, *p <* .001** |
| H3: Football ID → Match-Rel. Soc. | **RS; *b =* 0.565, *p <* .001** | **RS; *b =* 0.537, *p <* .001** | **RS; *b =* 0.615, *p <* .001** | **RS; *b =* 0.575, *p <* .001** | **RS; *b =* 0.486, *p <* .001** | **RS; *b =* 0.584, *p <* .001** |
| H3: National ID → Soc. Connect. | **RS; *b =* 0.201, *p <* .001** | **RS; *b =* 0.169, *p <* .001** | **RS; *b =* 0.230, *p <* .001** | **RS; *b =* 0.178, *p <* .001** | **RS; *b =* 0.211, *p <* .001** | **RS; *b =* 0.215, *p <* .001** |
| H3: National ID → Match-Rel. Soc. | **RS; *b =* 0.352, *p <* .001** | **RS; *b =* 0.342, *p <* .001** | **RS; *b =* 0.378, *p <* .001** | **RS; *b =* 0.343, *p <* .001** | **RS; *b =* 0.321, *p <* .001** | **RS; *b =* 0.369, *p <* .001** |
| H4: Soc. Connect. → Life Sat. | **RS; *b =* 0.286, *p <* .001** | **RS; *b =* 0.276, *p <* .001** | **RS; *b =* 0.258, *p <* .001** | **RS; *b =* 0.294, *p <* .001** | **RS; *b =* 0.308, *p <* .001** | **RS; *b =* 0.294, *p <* .001** |
| H4: Soc. Connect. → Work Motiv. | **RS; *b =* 0.333, *p <* .001** | **RS; *b =* 0.340, *p <* .001** | **RS; *b =* 0.304, *p <* .001** | **RS; *b =* 0.309, *p <* .001** | **RS; *b =* 0.381, *p <* .001** | **RS; *b =* 0.334, *p <* .001** |
| H4: Match-Rel. Soc. → Life Sat. | **RS; *b =* 0.171, *p <* .001** | **RS; *b =* 0.163, *p <* .001** | **RS; *b =* 0.158, *p <* .001** | **RS; *b =* 0.172, *p <* .001** | **RS; *b =* 0.188, *p <* .001** | **RS; *b =* 0.176, *p <* .001** |
| H4: Match-Rel. Soc. → Work Motiv. | **RI; *b =* 0.203, *p <* .001** | **RI; *b =* 0.185, *p <* .001** | **RI; *b =* 0.215, *p <* .001** | **RI; *b =* 0.195, *p <* .001** | **RI; *b =* 0.219, *p <* .001** | **RI; *b =* 0.202, *p <* .001** |
| H5a: Soc. Context x Result → Life Sat. [social context] | RI; *b =* 0.000, *p =* .999 | RI; *b =* 0.032, *p =* .676 | RI; *b =* -0.009, *p =* .891 | RI; *b =* 0.045, *p =* .488 | RI; *b =* -0.039, *p =* .617 | RI; *b =* -0.032, *p =* .626 |
| H5a: Soc. Context x Result → Life Sat. [result] | **RI; *b =* 0.418, *p <* .001** | **RI; *b =* 0.395, *p <* .001** | **RI; *b =* 0.404, *p <* .001** | **RI; *b =* 0.430, *p <* .001** | **RI; *b =* 0.435, *p <* .001** | **RI; *b =* 0.417, *p <* .001** |
| H5a: Soc. Context x Result → Life Sat. [interaction] | RI; *b =* 0.311, *p =* .103 | RI; *b =* 0.170, *p =* .458 | RI; *b =* 0.247, *p =* .225 | RI; *b =* 0.280, *p =* .156 | **RI; *b =* 0.622, *p =* .019** | RI; *b =* 0.317, *p =* .098 |
| H5b: Soc. Context x Result → Work Motiv. [social context] | **RI; *b =* -0.370, *p <* .001** | **RI; *b =* -0.406, *p =* .002** | **RI; *b =* -0.313, *p =* .007** | **RI; *b =* -0.401, *p <* .001** | **RI; *b =* -0.339, *p =* .005** | **RI; *b =* -0.372, *p <* .001** |
| H5b: Soc. Context x Result → Work Motiv. [result] | **RI; *b =* 0.354, *p <* .001** | **RI; *b =* 0.289, *p =* .002** | **RI; *b =* 0.304, *p <* .001** | **RI; *b =* 0.374, *p <* .001** | **RI; *b =* 0.455, *p <* .001** | **RI; *b =* 0.354, *p <* .001** |
| H5b: Soc. Context x Result → Work Motiv. [interaction] | RI; *b =* -0.071, *p =* .816 | RI; *b =* -0.027, *p =* .941 | RI; *b =* -0.417, *p =* .213 | RI; *b =* 0.024, *p =* .940 | RI; *b =* 0.177, *p =* .652 | RI; *b =* -0.071, *p =* .819 |
| H5: Soc. Context x Result → Team ID [waga] | RI; *b =* 0.061, *p =* .361 | RI; *b =* 0.074, *p =* .362 | RI; *b =* 0.012, *p =* .874 | RI; *b =* 0.108, *p =* .115 | RI; *b =* 0.050, *p =* .553 | RI; *b =* 0.041, *p =* .553 |
| H5: Soc. Context x Result → Team ID [result] | **RI; *b =* 0.643, *p <* .001** | **RI; *b =* 0.683, *p <* .001** | **RI; *b =* 0.698, *p <* .001** | **RI; *b =* 0.623, *p <* .001** | **RI; *b =* 0.544, *p <* .001** | **RI; *b =* 0.642, *p <* .001** |
| H5: Soc. Context x Result → Team ID [interaction] | RI; *b =* 0.056, *p =* .782 | RI; *b =* -0.201, *p =* .408 | RI; *b =* 0.034, *p =* .879 | RI; *b =* 0.004, *p =* .986 | **RI; *b =* 0.577, *p =* .043** | RI; *b =* 0.058, *p =* .769 |
| H5: Soc. Context x Result → Football ID [waga] | RI; *b =* 0.049, *p =* .426 | RI; *b =* 0.076, *p =* .317 | RI; *b =* 0.022, *p =* .754 | RI; *b =* 0.083, *p =* .197 | RI; *b =* 0.003, *p =* .962 | RI; *b =* 0.044, *p =* .501 |
| H5: Soc. Context x Result → Football ID [result] | **RI; *b =* 0.453, *p <* .001** | **RI; *b =* 0.458, *p <* .001** | **RI; *b =* 0.476, *p <* .001** | **RI; *b =* 0.485, *p <* .001** | **RI; *b =* 0.366, *p <* .001** | **RI; *b =* 0.453, *p <* .001** |
| H5: Soc. Context x Result → Football ID [interaction] | RI; *b =* -0.033, *p =* .862 | RI; *b =* -0.172, *p =* .452 | RI; *b =* -0.101, *p =* .629 | RI; *b =* -0.040, *p =* .837 | RI; *b =* 0.317, *p =* .203 | RI; *b =* -0.034, *p =* .857 |
| H5: Soc. Context x Result → National ID [waga] | RI; *b =* -0.091, *p =* .169 | RI; *b =* -0.103, *p =* .200 | RI; *b =* -0.085, *p =* .249 | RI; *b =* -0.046, *p =* .505 | RI; *b =* -0.147, *p =* .071 | RI; *b =* -0.102, *p =* .143 |
| H5: Soc. Context x Result → National ID [result] | **RI; *b =* 0.178, *p <* .001** | **RI; *b =* 0.203, *p <* .001** | **RI; *b =* 0.201, *p <* .001** | **RI; *b =* 0.156, *p =* .001** | **RI; *b =* 0.140, *p =* .012** | **RI; *b =* 0.178, *p <* .001** |
| H5: Soc. Context x Result → National ID [interaction] | RI; *b =* 0.247, *p =* .223 | RI; *b =* 0.036, *p =* .883 | RI; *b =* 0.085, *p =* .701 | RI; *b =* 0.258, *p =* .222 | **RI; *b =* 0.779, *p =* .005** | RI; *b =* 0.246, *p =* .228 |
| EXPL: Social Context → Alcohol | **RS; *b =* 0.917, *p <* .001** | **RS; *b =* 1.012, *p <* .001** | **RS; *b =* 0.842, *p <* .001** | **RS; *b =* 0.953, *p <* .001** | **RS; *b =* 0.745, *p <* .001** | **RS; *b =* 1.009, *p <* .001** |
| EXPL: Alcohol → Work Motiv. | **RI; *b =* -0.157, *p <* .001** | **RI; *b =* -0.172, *p <* .001** | **RI; *b =* -0.139, *p <* .001** | **RI; *b =* -0.175, *p <* .001** | **RI; *b =* -0.142, *p <* .001** | **RI; *b =* -0.159, *p <* .001** |
| Game Result → Life Sat. | **RS; *b =* 0.427, *p <* .001** | **RS; *b =* 0.400, *p <* .001** | **RS; *b =* 0.420, *p <* .001** | **RS; *b =* 0.438, *p <* .001** | **RS; *b =* 0.454, *p <* .001** | **RS; *b =* 0.427, *p <* .001** |
| Game Result → Work Motiv. | **RI; *b =* 0.358, *p <* .001** | **RI; *b =* 0.283, *p =* .002** | **RI; *b =* 0.298, *p <* .001** | **RI; *b =* 0.385, *p <* .001** | **RI; *b =* 0.478, *p <* .001** | **RI; *b =* 0.358, *p <* .001** |
| Game Result → Team ID | **RS; *b =* 0.662, *p <* .001** | **RS; *b =* 0.705, *p <* .001** | **RS; *b =* 0.721, *p <* .001** | **RS; *b =* 0.632, *p <* .001** | **RS; *b =* 0.587, *p <* .001** | **RS; *b =* 0.658, *p <* .001** |
| Game Result → Football ID | **RS; *b =* 0.465, *p <* .001** | **RS; *b =* 0.464, *p <* .001** | **RS; *b =* 0.492, *p <* .001** | **RS; *b =* 0.491, *p <* .001** | **RS; *b =* 0.392, *p <* .001** | **RS; *b =* 0.471, *p <* .001** |
| Game Result → National ID | **RS; *b =* 0.184, *p <* .001** | **RS; *b =* 0.204, *p =* 0.001** | **RS; *b =* 0.204, *p <* .001** | **RS; *b =* 0.162, *p =* 0.003** | **RS; *b =* 0.172, *p =* .005** | **RS; *b =* 0.179, *p <* .001** |
| Game Result → Soc. Connect. | **RS; *b =* 0.406, *p <* .001** | **RS; *b =* 0.369, *p <* .001** | **RS; *b =* 0.407, *p <* .001** | **RS; *b =* 0.378, *p <* .001** | **RS; *b =* 0.477, *p <* .001** | **RS; *b =* 0.407, *p <* .001** |
| Game Result → Match-Rel. Soc. | **RS; *b =* 1.028, *p <* .001** | **RS; *b =* 1.050, *p <* .001** | **RS; *b =* 1.121, *p <* .001** | **RS; *b =* 1.102, *p <* .001** | **RS; *b =* 0.808, *p <* .001** | **RS; *b =* 1.029, *p <* .001** |
| **Number of models with same direction** | **35 out of 41 significant models** | | | | | |
| **Number of models with similar significance** | **38 out of 41 models** | | | | | |

*Note.* Significant results are displayed in bold. RI = Random-intercept model; RS = Random-intercept random-slope model. The better-fitting 2-level model (RI vs. RS, based on χ² test, *p <* .05) is reported alongside the corresponding leave-one-out model (leaving one country out).

**Table S16**

*Results of Main and Robustness Analysis for Detailed Review of Game Result Variable Including All Games vs. Only Games Followed by a Weekday*

| **Path** | **Main Analysis (All Games)** | **Robustness Analysis (Weekday Games Only)** |
| --- | --- | --- |
| **H1: Social Context → Outcomes** | | |
| H1a: Social Context → Life Satisfaction | RI; *b* = -0.005, *p* = .934 | RI; *b* = -0.027, *p* = .775 |
| H1b: Social Context → Work Motivation | **RI; *b* = -0.382, *p <* .001** | RI; *b* = -0.045, *p* = .751 |
| **H2: Social Context → Social Identities & Social Processes** | | |
| H2a: Social Context → Team Identity | RI; *b* = 0.044, *p* = .538 | RI; *b* = -0.011, *p* = .917 |
| H2b: Social Context → Football Identity | RI; *b* = 0.035, *p* = .581 | RI; *b* = 0.017, *p* = .868 |
| H2c: Social Context → National Identity | RI; *b* = -0.091, *p* = .171 | RI; *b* = -0.115, *p* = .270 |
| H2d: Social Context → Social Connectedness | RI; *b* = 0.062, *p* = .417 | RI; *b* = -0.043, *p* = .703 |
| H2e: Social Context → Match-Rel. Socializing | RI; *b* = -0.030, *p* = .774 | RI; *b* = -0.314, *p* = .064 |
| **H3: Social Identities → Social Processes** | | |
| H3a: Team Identity → Social Connectedness | **RI; *b* = 0.316, *p <* .001** | **RI; *b* = 0.356, *p <* .001** |
| H3b: Team Identity → Match-Rel. Socializing | **RI; *b* = 0.503, *p <* .001** | **RI; *b* = 0.697, *p <* .001** |
| H3c: Football Identity → Social Connectedness | **RI; *b* = 0.286, *p <* .001** | **RI; *b* = 0.326, *p <* .001** |
| H3d: Football Identity → Match-Rel. Socializing | **RI; *b* = 0.576, *p <* .001** | **RI; *b* = 0.727, *p <* .001** |
| H3e: Country Identity → Social Connectedness | **RI; *b* = 0.225, *p <* .001** | **RI; *b* = 0.303, *p <* .001** |
| H3f: Country Identity → Match-Rel. Socializing | **RI; *b* = 0.354, *p <* .001** | **RI; *b* = 0.380, *p <* .001** |
| **H4: Social Processes → Outcomes** | | |
| H4a: Social Connectedness → Life Satisfaction | **RI; *b* = 0.313, *p <* .001** | **RI; *b* = 0.315, *p <* .001** |
| H4b: Social Connectedness → Work Motivation | **RI; *b* = 0.343, *p <* .001** | **RI; *b* = 0.398, *p <* .001** |
| H4c: Match-Rel. Socializing → Life Satisfaction | **RI; *b* = 0.183, *p <* .001** | **RI; *b* = 0.205, *p <* .001** |
| H4d: Match-Rel. Socializing → Work Motivation | **RI; *b* = 0.203, *p <* .001** | **RI; *b* = 0.273, *p <* .001** |
| **H5: Moderation — Social Context × Game Result** | | |
| H5a: Soc. Context × Result → Life Satisfaction [social context] | RI; *b* = 0.000, *p* = .999 | RI; *b* = 0.019, *p* = .835 |
| H5a: Soc. Context × Result → Life Satisfaction [result] | **RI; *b* = 0.418, *p <* .001** | **RI; *b* = 0.457, *p <* .001** |
| H5a: Soc. Context × Result → Life Satisfaction [interaction] | RI; *b* = 0.311, *p* = .103 | RI; *b* = 0.076, *p* = .763 |
| H5b: Soc. Context × Result → Work Motivation [social context] | **RI; *b* = -0.370, *p <* .001** | RI; *b* = 0.037, *p* = .781 |
| H5b: Soc. Context × Result → Work Motivation [result] | **RI; *b* = 0.354, *p <* .001** | **RI; *b* = 0.739, *p <* .001** |
| H5b: Soc. Context × Result → Work Motivation [interaction] | RI; *b* = -0.071, *p* = .816 | RI; *b* = -0.140, *p* = .700 |
| H5c: Soc. Context × Result → Team Identity [social context] | RI; *b* = 0.061, *p* = .360 | RI; *b* = 0.054, *p* = .590 |
| H5c: Soc. Context × Result → Team Identity [result] | **RI; *b* = 0.643, *p <* .001** | **RI; *b* = 0.675, *p <* .001** |
| H5c: Soc. Context × Result → Team Identity [interaction] | RI; *b* = 0.056, *p* = .781 | RI; *b* = 0.191, *p* = .486 |
| H5d: Soc. Context × Result → Football Identity [social context] | RI; *b* = 0.049, *p* = .426 | RI; *b* = 0.069, *p* = .473 |
| H5d: Soc. Context × Result → Football Identity [result] | **RI; *b* = 0.453, *p <* .001** | **RI; *b* = 0.513, *p <* .001** |
| H5d: Soc. Context × Result → Football Identity [interaction] | RI; *b* = -0.033, *p* = .862 | RI; *b* = 0.057, *p* = .833 |
| H5e: Soc. Context × Result → National Identity [social context] | RI; *b* = -0.091, *p* = .168 | RI; *b* = -0.104, *p* = .317 |
| H5e: Soc. Context × Result → National Identity [result] | **RI; *b* = 0.178, *p <* .001** | **RI; *b* = 0.154, *p* = .015** |
| H5e: Soc. Context × Result → National Identity [interaction] | RI; *b* = 0.247, *p* = .223 | RI; *b* = 0.178, *p* = .541 |
| **EXPL: Alcohol Consumption as Mediator** | | |
| EXPL: Social Context → Alcohol Consumption | **RI; *b* = 0.855, *p <* .001** | **RI; *b* = 0.807, *p <* .001** |
| EXPL: Alcohol Consumption → Work Motivation | **RI; *b* = -0.157, *p <* .001** | **RI; *b* = -0.124, *p <* .001** |
| **EXPL: Game Result → Outcomes, Identities & Social Processes** | | |
| EXPL: Game Result → Life Satisfaction | **RI; *b* = 0.422, *p <* .001** | **RI; *b* = 0.458, *p <* .001** |
| EXPL: Game Result → Work Motivation | **RI; *b* = 0.358, *p <* .001** | **RI; *b* = 0.735, *p <* .001** |
| EXPL: Game Result → Team Identity | **RI; *b* = 0.643, *p <* .001** | **RI; *b* = 0.677, *p <* .001** |
| EXPL: Game Result → Football Identity | **RI; *b* = 0.452, *p <* .001** | **RI; *b* = 0.512, *p <* .001** |
| EXPL: Game Result → Country Identity | **RI; *b* = 0.183, *p <* .001** | **RI; *b* = 0.162, *p* = .010** |
| EXPL: Game Result → Social Connectedness | **RI; *b* = 0.397, *p <* .001** | **RI; *b* = 0.326, *p <* .001** |
| EXPL: Game Result → Match-Rel. Socializing | **RI; *b* = 1.065, *p <* .001** | **RI; *b* = 1.297, *p <* .001** |
| **Number of models with same direction** | **38 out of 41 models** |  |
| **Number of models with similar significance** | **39 out of 41 models** |  |

*Note.* Significant results are displayed in bold. RI = Random-intercept model. All models are random-intercept only; random-intercept random-slope models are unidentifiable for the weekday games dataset (insufficient within-person variance) and are therefore not reported.
